# Organ cross-talk: molecular mechanisms, biological functions, and therapeutic interventions for diseases

**DOI:** 10.1038/s41392-025-02329-1

**Published:** 2026-01-06

**Authors:** Huiting Che, Yidan Gao, Yonghu Xu, Hui Xu, Roland Eils, Mei Tian

**Affiliations:** 1 Human Phenome Institute, Fudan University, Shanghai, China; 2 Department of Gastric Surgery, Fudan University Shanghai Cancer Center, Shanghai, China; 3 Center for Digital Health, Berlin Institute of Health at Charité—Universitätsmedizin Berlin, Berlin, Germany; 4 Intelligent Medicine Institute, Fudan University, Shanghai, China

**Keywords:** Physiology, Cell biology, Diseases

## Abstract

Organ cross-talk, also known as the organ axis or organ interaction network, plays a vital role in maintaining physiological homeostasis and responding to environmental stimuli. This review comprehensively integrates cutting-edge observations in organ communication research, with a particular focus on the brain, heart, and gut—the three core organs that garner the most attention in organ connection studies. The current state of organ interaction network research is clearly presented as a Sankey diagram. For brain-related connections, the interactions among the brain-gut, brain-liver, and brain-heart connections are thoroughly reviewed; for heart-related connections, the relationships among the heart–kidney, heart–lung, and heart–liver connections are explored in detail; and for gut-related connections, the interactions among the gut–liver, gut–kidney, and gut–lung connections are emphasized. Additional information on other prevalent organ connections is systematically organized in tables for intuitive presentation. Through the integration of profound insights into molecular mechanisms and biological functions, the complex signaling pathways regulating organ interactions in health and disease states have been systematically elucidated. In terms of therapeutic strategy development, numerous directions with potential application value are proposed on the basis of these research findings. Furthermore, this review meticulously discusses the diverse methods and advanced technologies employed in organ connection research, comprehensively highlighting the critical role of technological support in advancing this field. In the future, this review advocates the adoption of network-driven models, innovative diagnostic approaches, and personalized treatment strategies to offer new perspectives for addressing complex diseases from a systems biology standpoint.

## Introduction

Organ cross-talk research stands at the forefront of life sciences because of its critical role in understanding physiological homeostasis and disease mechanisms.^1–5^ In this study, “organ” refers to a collection of tissues joined in a structural unit to serve a common function, exemplified by the brain, heart, and gut.^6^ Organ cross-talk refers to complex biological communication and feedback between different organs and is mediated by mechanical, soluble, and cellular mechanisms, which are essential for maintaining body homeostasis but can result in functional and structural dysfunction in other organs when pathological states occur in one or more organs.^7^ Traditional medical approaches, which emphasize individual organs through anatomy, physiology, and pathology, have offered valuable insights but fail to address the dynamic interplay among organs. In contrast, organ cross-talk research adopts a systems biology perspective, highlighting the significance of interorgan collaboration in health and disease.^8,9^

The study of organ cross-talk often begins with disease models. For example, in pulmonary heart disease, chronic lung pathology increases right ventricular afterload, leading to heart failure, exemplifying the lung‒heart axis’s pathological interaction.^10^ Similarly, in hepatorenal syndrome, liver dysfunction disrupts renal filtration through inflammatory mediators and metabolic toxicity, underscoring the complexity of liver–kidney cross-talk.^11^ Beyond disease contexts, basic medical research has illuminated organ connections in health, such as the gut‒brain axis, which regulates emotion and cognition via the vagus nerve and microbial metabolites.^12^ Moreover, the gut‒liver‒brain axis exemplifies a three-way interaction system in which the gastrointestinal tract, liver, and nervous system communicate through gut microbial metabolites, immune responses, and neural signaling, influencing both metabolic homeostasis and neurological health.^13^ These findings demonstrate that complex interorgan interactions are essential for maintaining physiological balance and overall health, with networks involving three or more organs further deepening our understanding of human physiological complexity.

Recent advances in multiomics, artificial intelligence (AI), and imaging technologies have significantly propelled this field forward.^14,15^ For example, a study of 30,444 UK Biobank (UKB) participants using multiorgan magnetic resonance imaging (MRI) phenotypes revealed direct and indirect pathways within the heart–brain–liver axis, whereas molecular imaging and computational techniques have constructed comprehensive frameworks of metabolic connectivity.^16^ These discoveries underscore the transformative potential of emerging technologies in unraveling complex physiological processes and driving medical innovation.

This article systematically reviews the research hotspots in organ cross-talk in health and disease, elucidating the signaling pathways and mechanisms involving the brain, heart, and gut, thereby providing a theoretical and practical foundation for advancing studies on disease progression and novel therapeutic strategies. Given the scope of this review, the discussion focuses primarily on brain, heart, and gut interactions, with other organs and dynamic network analyses explored in future research.

## Current status of organ connection research

**Fig. 1:**
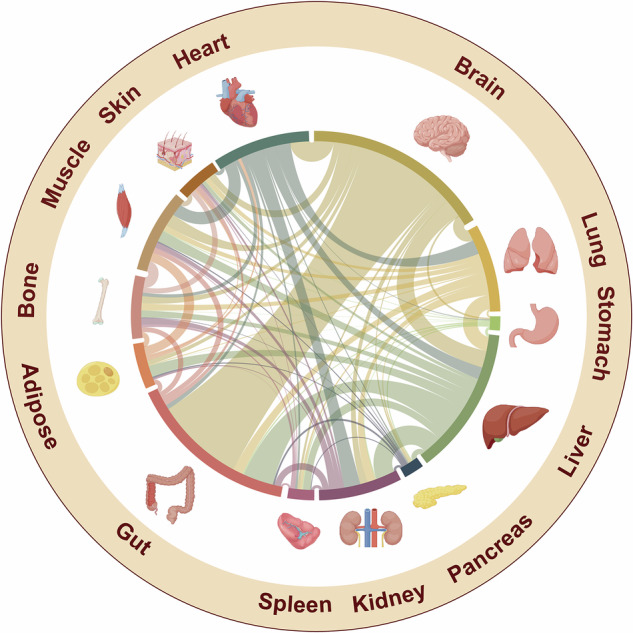
Sankey diagram presenting the current state of organ cross-talk research from 2015 to 2025. The thickness of the connecting chords represents the number of articles related to each organ pair, with prominent connections (e.g., brain–gut, gut-liver, liver–kidney) indicating high research intensity, whereas thinner links (e.g., stomach-pancreas) reflecting less attention. This diagram offers a comprehensive overview of the field, highlighting key focus areas and the dynamic interplay between organs. The figure was created by Figdraw (www.figdraw.com)

This section explores the landscape of organ cross-talk research, highlighting key hotspots, mechanistic insights into the three most studied organs, and their implications for disease intervention. By integrating diverse studies, we illuminate the current understanding of interorgan communication and the critical role of signal transduction in these dynamic interactions. To map the prominence of organ cross-talk studies, we searched PubMed (2015–2025) via paired organ terms with keywords such as “cross-talk,” “axis,” or “network,” identifying 50,523 relevant articles. These findings are presented in a Sankey diagram (Fig. 1) and detailed in tables (Tables 1 and 2), highlighting research trends, dominant organ networks, and therapeutic opportunities.

**Table 1:** Overview of interorgan cross-talk

Organ	Cross-talk partner	Potential mechanisms
**Brain**	**Heart**	CNS controls cardiovascular activity by sending signals through the autonomic nervous system to the cardiac conduction system and myocardium via the sympathetic and parasympathetic pathways^118^ An altered autonomic nervous system signaling and increased systemic inflammation in PTSD and CVD.^120^ Certain brain–heart disorders, such as Takotsubo syndrome, can be reversed by estrogen/progesterone supplementation.^413^
	**Lung**	Systemic inflammatory environment in brain injury (first hit) leads to extraversion of fluid in the alveolar and interstitial space which triggers lung injury (second hit).^413^ Systemic and pulmonary pressures could return to normal values in some reports, yet the capillary-alveolar membrane damage remains.^414^
	**Liver**	Signals from the liver control eating behavior in the CNS. Neural signals from the CNS affect glucose, lipid, and protein metabolism in the liver.^415^ Disorders in the brain–liver can be transient and reversible by addressing the underlying liver dysfunction, such as detoxification.^416^
	**Kidney**	Activation of the HPA axis, SNS, and RAAS following a stroke can disrupt hormone and neurotransmitter release, potentially leading to impaired kidney function.^417,418^ Reversibility of the brain-kidney cross-talk is demonstrated by CKD mouse models, which exhibit improved working memory after treatment with tempol, an antioxidant.^419^
	**Pancreas**	Intracerebroventricular administration of FGF21 regulates blood glucose levels by enhancing insulin metabolism and changing glucose utilization.^420^
	**Spleen**	A neuroinflammatory environment induced by ischemic stroke activate spleen-mediated peripheral immune response. Release of inflammatory mediators pass through the leaky blood brain barrier, further exacerbating brain injury.^421^
	**Stomach**	The brain and stomach are thought to be connected by the conventional regulation of energy balance of and the gastric intracellular mammalian target of rapamycin (mTOR)/S6K1 pathways involved in ghrelin, nesfatin, endocannabinoid metabolisms.^422^
	**Gut**	Toxin induced signals are transmitted through both the immune and neuroendocrine pathways within the gut, which signals distinct brainstem circuits that drive nausea and retching.^39^ The brain–gut cross-talk has been highlighted in depression, anxiety, schizophrenia, epilepsy, and AD both from animal and clinical studies.^72,423^ Strategies that involved in ameliorating the aforementioned disorders focus on the mediation of microbial composition through probiotics, dysbiosis reversal through antibiotics, or direct supplementation of microbial metabolites through prebiotics.^423^
	**Adipose**	The CNS plays vital role in directing the differentiation of adipose tissues to brown, white, and beige adipose tissues by signaling through the sympathetic regulation of leptin production during fasting and satiety.^424^
	**Bone**	The brain impacts bone function via the secretion of molecules, involving neurohormones (growth hormones, oxytocin, etc.), neuropeptides (agouti-related peptide, proopiomelanocortin, etc.) and neurotransmitters (acetylcholine, dopamine, etc.). Bone-derived mediators from bone cells and bone marrow, such as osteocalcin, lipocalin-2, receptor activator of NF-κB ligand can in turn influence brain function.^425^ Denosumab, a RANKL-neutralizing monoclonal antibody has shown effect in improving bone loss and depression.^426^
	**Muscle**	Sarcopenia decreases gait velocity and cognitive function.^427^ Exercise is a good remedy for disorders of the brain-muscle cross-talk. It improves neurological functions in two ways: Skeletal source of PGC-1α1 during exercise changes kynurenine metabolism and alleviate stress-induced depression or fatigue^428,429^; Increased level of brain-derived neurotrophic factor during physical activity improves cognitive performances.
	**Skin**	The brain-skin axis describes the bidirectional communication between the brain and skin, involving neural, endocrine, and immune pathways. Psychological stress activates the HPA axis, releasing cortisol and proinflammatory cytokines, which exacerbate conditions like psoriasis by disrupting skin barrier function and immune responses.^430,431^ Additionally, stress-induced neuropeptides, such as substance P, further amplify inflammation and skin cell proliferation, highlighting the intricate link between mental health and skin health.^431^
**Heart**	**Lung**	The heart and lungs are intimately connected both anatomically and functionally, jointly responsible for blood oxygenation and the maintenance of systemic circulation. Clinically, common pulmonary conditions, such as COPD or pulmonary hypertension, significantly increase the afterload on the right ventricle, potentially leading to right ventricular failure, also known as cor pulmonale. IPF is another condition where complications can include congestive heart failure.^432^ Heart failure is marked by pathological hemodynamic disturbances. In the lungs, these disturbances result in dysfunctions in ventilatory control and efficiency, pulmonary congestion, capillary pressure imbalances, and can ultimately contribute to the progression of pulmonary vascular disease.^161^
	**Liver**	PET/CT imaging study concludes that coronary microvascular dysfunction occurs more frequently in patients with MAFLD.^433^ T1-MRI identified liver fibrosis is found to associated with a history of HF, atrial fibrillation, and coronary heart disease.^434^
	**Kidney**	The interactions between the cardiovascular and renal systems are characterized by the balance of extracellular fluid volume and blood pressure regulation. In cardiorenal syndrome, which involves the heart–kidney cross-talk, decreased cardiac output and blood pressure trigger simultaneous activation of the SNS and RAAS. The negative feedback loop promotes sodium and water retention, exacerbating HF by reducing arterial pressure and increasing renal venous pressure.^435^ The implementation of a left ventricular assist device (LVAD) has the potential to reverse the adverse effects of HF on the kidneys (characterized by increased renal perfusion rate), although prognosis is dependent on distinct patient management strategies.^436^
	**Pancreas**	Pancreatic dysfunction can lead to diabetes, which in turn increases the risk of a range of cardiovascular complications, with HF being one of the most severe outcomes.^437^
	**Stomach**	Ghrelin, a growth hormone-releasing peptide first identified in the stomach, plays a role in stimulating growth hormone release and appetite. It has been shown to have beneficial effects on the heart, including improving cardiac function and remodeling, alleviating symptoms of HF, and enhancing survival rates following myocardial infarction. The mechanisms underlying these effects may involve both direct actions on cardiovascular cells and modulation of autonomic nervous system activity.^438^
	**Gut**	Different degrees of HF (including those requiring LVAD, or heart transplantation) are related to gut dysbiosis (decrease in alpha diversity), inflammation and endotoxemia.^439^ Its mechanism is explained by the bowel wall edema and regional hypoxia triggered by HF, which leads to gut microbiome alteration. Microbial components such as LPS, metabolites, such as TMAO, also contributes to HF. Prebiotics, probiotics, dietary style that alter gut immunity, physical activity have been found to alleviate cardiovascular complications in human studies.
	**Adipose**	The heart and the adipose tissue are connected and regulated via the SNS. β-adrenergic receptor signaling mediates lipolysis in adipose tissue and regulates heart rate and force of contraction. In addition, natriuretic peptides (NPs) released from the heart triggers brown fat differentiation and thermogenesis.^440,441^ Obesity and insulin resistance prevent natriuretic peptide production in the heart, interfering with the mode of energy expenditure.^152^
	**Bone**	The interaction between the myocardium and bone marrow (BM) after acute myocardial infarction is mediated by canonical Wnt signaling in the BM, which leads to BM progenitor cell proliferation and mobilization to circulation.^442^
	**Muscle**	Both anabolic factors, such as growth hormone and insulin-like growth factor-1 and catabolic factors, such as TNF, IL-1β, IFN-γ, myostatin, are involved in maintaining muscle structure and function.^443^ Exercise capacity of HF patients is primarily determined by their skeletal muscle mass and muscle strength. In HF, the reduced peak oxygen uptake leads to histological abnormalities, changes in mitochondrial structure and function, oxidative stress, and a shift in muscle fiber distribution. Exercise training with an aim to boost muscle capacity and strength has shown potential in reversing muscle wasting in HF.^444^
**Lung**	**Liver**	Impaired hepatic blood flow or compromised hepatic cell integrity during sepsis adversely induces lung inflammation and diminish the overall performance of extrahepatic organs in sepsis.^445^
	**Kidney**	Certain mechanisms that contribute to the onset and advancement of CKD related to the lung involves vascular stiffness, activation of neurohormonal pathways, tissue hypoxia, respiratory acidosis, impaired gas exchange, systemic congestion, respiratory support/replacement therapies and dysregulated immune cell signaling.^446^ Noninvasive ventilation, conservative fluid management and early management of pulmonary infections show promise as treatment approaches for addressing impaired renal microcirculation.^447^
	**Pancreas**	Pancreatic dysfunction can lead to diabetes, which in turn exacerbates lung diseases such as COPD and respiratory infections.^448^ Diabetes patients are at higher risk for respiratory viral infections, including influenza and SARS-CoV-2.^449^
	**Stomach**	People with gastroesophageal reflux disease (GERD) have a higher risk of developing COPD, bronchitis, pneumonia, lung cancer, and pulmonary embolism.^450^
	**Gut**	The communities found in both the intestinal and airway microbiotas consist of diverse populations, with the phyla *Bacteroidetes* and *Firmicutes* being the most prevalent.^451^ A low gut microbial diversity during early infancy is highly associated with development of childhood asthma.^452^
	**Adipose**	Obesity may play a significant role in the pathogenesis of pulmonary diseases through proinflammatory mediators produced by adipose tissue, which contribute to a state of systemic low-grade inflammation.^453^ In animal models, inflammatory responses in the lung have been shown to influence the production of adipocytokines (such as leptin and adiponectin), cytokines, acute phase proteins, and other mediators produced by adipose tissue that may participate in immune responses of the lung.^453^ An increased adipose tissue mass may also affect susceptibility to pulmonary infections, enhance pulmonary inflammation associated with environmental exposures, and exacerbate airway obstruction in preexisting lung disease.^454^
	**Muscle**	Muscle dysfunction is a common systemic manifestation in COPD and has a significant impact on exercise tolerance, thereby worsening patients’ quality of life and survival. Oxidative stress is one of the main causes of skeletal muscle dysfunction. Specifically, protein carbonylation, a common form of protein oxidation, has been shown to alter the function of key enzymes and structural proteins involved in muscle contractile performance.^455^
**Liver**	**Kidney**	Fibrotic MAFLD increases the risk of CKD, indicated by reduced estimated GFR. In addition, decreased level of leptin is found to be an independent predictor for CKD risk.^456^
	**Pancreas**	In hepatic insulin resistance mouse models, an endocrine axis between the liver and pancreas has been investigated, wherein insulin-like growth factor-1 (IGF-1), originated from the liver, promotes compensatory hyperplasia of pancreatic islets through the activation of insulin receptor isoform A.^457^
	**Spleen**	mTOR pathway is overactivated in spleen during liver cirrhosis, leading to overexuberant lymphocyte proliferation, which can be readily inhibited by mTOR inhibitor rapamycin. For other aspects of liver related disease, excellent reviews can be referred.^458,459^
	**Stomach**	The liver and stomach both belong to the digestive system. Liver diseases, such as cirrhosis, can impact gastrointestinal function, leading to abnormal gastric acid secretion and potentially causing gastric discomfort and digestive issues. Impaired liver function may also affect the normal secretion of bile, which in turn impacts digestive function in the stomach. Additionally, liver diseases like cirrhosis can lead to portal hypertension, which may cause esophageal varices and increase the risk of gastrointestinal bleeding.^460,461^
	**Gut**	Bilirubin, produced by the liver, is related to the liver–gut cross-talk as it undergoes conversion by the gut microbiota into urobilinogen within the intestine.^376^ Microplastic ingestion in mouse model has led to increased liver–gut cross-talk that eventually contributes to insulin resistance and diabetes.^462^
	**Adipose**	Thermogenesis in adipose tissue is partly governed by hepatic small ubiquitin-related modifier (SUMO)-specific protease 2 (SENP2).^463^ In another mouse study, brown and beige adipose tissue thermogenesis can antagonize liver inflammation in MAFLD.^358^ In addition, the liver plays a crucial role in sensing various metabolic states and communicates with adipose tissue to facilitate adaptive changes in response to lipid overload.^464^
	**Bone**	Hepatokines, such as FGF21, Bone morphogenetic protein 9 (BMP9) and osteokines, such as osteocalcin, calcitonin, are found to act as bridges between liver and bone, which are highlighted in many metabolic and bone diseases.^465^
	**Muscle**	Liver and muscle complications share common pathophysiological pathways, including insulin resistance, myokine metabolism, hyperammonemia. An example is that the imbalance between phosphatidylinositol 3-kinase/Akt and myostatin signaling pathways leads to muscle wasting in MAFLD-related fibrosis.^466^
**Kidney**	**Pancreas**	Young patients with type 2 diabetes exhibit severe β-cell dysfunction and insulin resistance, leading to increased intraglomerular pressure and elevated urine albumin-to-creatinine ratio, which in turn causes kidney damage. Additionally, these patients show relative hyperoxia in the kidneys, indicating that insulin dysfunction is closely linked to changes in renal hemodynamics and oxygen supply, thereby amplifying the risk of kidney injury.^182^
	**Stomach**	Uncommon mucosal alterations in the stomach and duodenum of a kidney transplant recipient following prolonged peritoneal dialysis.^467^ Gastric *Helicobacter pylori* infection may increase the risk of CKD.^468^
	**Gut**	Mouse models for proteinuric kidney injury exhibit notable alterations in the composition, structure, and function of intestinal lymphatics, characterized by enhanced lymphangiogenesis, increased lymph flow, and augmented transport of lipoproteins and proinflammatory substances.^469,470^
	**Adipose**	Adipose tissue secretes various hormones and cytokines, such as leptin, adiponectin, and growth factors (e.g., insulin-like growth factor-1, IGF-1). These secretions can influence kidney function through the bloodstream.^471^ Kidney dysfunction (e.g., CKD) may affect lipid metabolism, leading to lipid accumulation in the body, which can exacerbate obesity and metabolic syndrome.^174,175^
	**Bone**	The bone-derived hormone FGF23 works with parathyroid hormone (PTH) and active vitamin D (1,25D) to regulate phosphate and calcium balance. When blood levels of phosphate and 1,25D rise, bone tissue produces FGF23. FGF23 promotes phosphate excretion and lowers 1,25D levels by binding to FGF receptors and the coreceptor α-Klotho in the kidney. Other biomolecules produced by the kidney, such as lipocalin-2 and erythropoietin, also influence FGF23 synthesis.^472^
	**Muscle**	Sarcopenia frequently accompanies CKD and fibrosis due to a gradual decrease of exercise-induced myokine, irisin. In addition, exosome and its associated microRNA are also found to be mediating this duo-cross-talk.^473^
**Pancreas**	**Stomach**	Instead of a mere coworker, the stomach influences the pancreas by mediating its fibrosis through the stimulation of mTORC1 activity in gastric X/A-like cells, eventually drives insulin resistance, as presented in mouse models of pancreatic fibrosis.^474^
	**Gut**	Pancreas is involved not only in the secretion of digestive enzymes and hormones but also in the maintenance of intestinal balance and control of inflammation, including the protection of the intestinal mucosa against pathogens and pathobionts.^475^
	**Adipose**	Pancreatic fat is associated with reduced insulin secretion, particularly in specific situations such as prediabetes, low BMI, and increased genetic risk of type 2 diabetes.^476^
	**Bone**	VThere is a positive correlation between insulin resistance and osteoporosis. Research has found that there is an interaction between the insulin signaling pathway and the Wingless (Wnt)/β-catenin signaling pathway in bone homeostasis.^477^ Bone cells not only function within the local bone environment but also regulate insulin secretion in the pancreas through their endocrine role.^478^
	**Muscle**	Insulin regulates glucose uptake in skeletal muscle through complex signaling pathways. After exercise or contraction, skeletal muscle increases its sensitivity to insulin, enhancing its ability to absorb glucose. This mechanism plays a crucial role in overall glycemic control. However, in cases of obesity and type 2 diabetes, the muscle’s response to insulin may be impaired, leading to reduced efficiency in glucose uptake.^479^
**Spleen**	**Gut**	Alginate oligosaccharide (AOS)-improved gut microbiota (A10-FMT) can repair spleen vasculature damage caused by busulfan. A10-FMT enhances cell proliferation rates, endothelial progenitor cell capabilities, and cell junction molecules, thereby increasing spleen vascularization.^480^ Depletion of circulating IgM memory B cells is associated with a defect in intestinal IgA-secreting plasma cells in asplenia and CVID. The unique role of IgM memory B cells in mucosal protection suggests the existence of a functional gut-spleen axis.^481^
	**Adipose**	High-fat diet significantly impacts the immune cell populations in the spleen. Studies have shown that HFD increases the frequency of CD3 + T cells and cNK cells in the spleen. At the same time, HFD decreases the frequency of NKp46+ NK cells in the spleen. These changes suggest that HFD may affect immune responses by modulating the distribution and phenotype of immune cells in the spleen.^482^
**Stomach**	**Gut**	The colonization of *Helicobacter pylori* significantly impacts the gastric microenvironment, which in turn affects the gastric microbiota and may be associated with changes in the colonic microbiota.^483^
	**Bone**	The regulation of gastric acid secretion and bone metabolism is highly interconnected and plays a crucial role in various diseases involving dysfunction of these organs or related mediators. This interaction is mediated by many endocrine modulators such as 1,25(OH)2 vitamin D (calcitriol), PTH, calcitonin and calcium.^484^
**Gut**	**Adipose**	The cross-talk between the gut and adipose tissue is mediated by the iron concentration within the adipocytes, which influence gut lipid absorption, as indicated in mouse models.^485,486^ Excellent review has been published to elucidate more details about such cross-talk.^487^
	**Bone**	Bone density, primarily managed through osteoclast and osteoblasts, can be influenced by microbiota through mechanisms such as calcium absorption, RANKL pathway, the regulation of serum levels of IGF-1 and the secretion of metabolites. Complementary therapeutic strategies that restore microbial homeostasis, such as dietary supplements, pre/probiotics, benefits bone health.^488,489^
	**Muscle**	The gut–muscle axis involves various biological mechanisms. The gut microbiota is regarded as a “metabolic organ” that can regulate muscle energy metabolism, protein synthesis, and mitochondrial function by producing metabolites such as SCFAs.^490^ Probiotic supplementation can optimize the composition of the gut microbiota, reduce age-related sarcopenia and oxidative stress, and enhance muscle function.^491^ Additionally, excessive training and poor diet may disrupt intestinal homeostasis, increase inflammation, and consequently affect muscle health.^492^
	**Skin**	The gut-skin axis highlights the connection between gut health and skin conditions through immune and metabolic pathways. Dysbiosis can lead to systemic inflammation, exacerbating issues like atopic dermatitis and food allergies, while gut-derived metabolites, such as SCFAs, help regulate skin health and aging. This interplay underscores the importance of gut microbiome balance in maintaining healthy skin.^493–495^
**Adipose**	**Bone**	Increasing evidence suggests that certain factors released from peripheral adipose tissue into the bloodstream may adversely affect bone quality, thereby increasing the risk of fractures. The local interaction between adipose tissue and bone within the bone marrow plays a significant role in the pathogenesis of age-related bone loss and osteoporosis. Bone marrow adipose tissue is considered an active component of the bone marrow niche, exerting both paracrine and endocrine functions that regulate osteogenesis and hematopoiesis.^496,497^
	**Muscle**	Myokines, such as irisin and IL-6, play a role in adipose tissue functions, such as lipolysis and browning. Adipokines, such as adipokines and adiponectin, participate in muscle metabolism by regulating glucose uptake, etc. Muscle tone and proportion changes as one age. Exercise and dietary supplement of flavonoid are two measures to strength adipose tissue-muscle cross-talk and enhancing health span.^498^
**Bone**	**Muscle**	Bone and muscle are connected via two mechanisms: mechanical loading and reciprocal soluble factors, including prostaglandin E2 (PGE2), Wnt3a, osteocalcin (OCN), IGF-1 and sclerostin.^499^

**Table 2:** Overview of an organ axis or network that involves three or more organs/systems

Organ	Partners involved	Potential mechanisms
**Brain**	**Heart** **Liver**	MRI derived features of the heart, liver and brain are found to correlate strongly. Certain features include liver fibro-inflammation, liver fat, aortic stiffening and left ventricle stroke volume are found to associate strongly with gray matter volume and white matter hyperintensities.^16^
	**Heart** **Kidney**	Pressure overload to the left ventricle activates sympathetic nerves that innervate the kidneys, which stimulates renal macrophage that feedback on the cardiac macrophages to switch to the cardiac hypertrophic program. Pharmacological modulation of β2-receptor, such as adrenergic β2-receptor blockade can affect the heart–brain–kidney network.^500^
	**Gut** **Kidney** **Immune**	Persistent neuroinflammation of the brain increases blood pressure and potentiates pathogenesis of the gut and kidney. Bone marrow derived immune cells activated by the gut microbiota enter the circulation, which triggers gut and renal inflammation, leads to dysbiosis and disorders in intestinal metabolism and impaired kidney function.^269^
	**Gut** **Pancreas**	The trio communicates via a series of hormones including glucagon-like peptide-1 and 2 (GLP-1/2), peptide YY, serotonin, and motilin etc.^269,501^.
	**Gut** **Immune**	The interplay between networks of immune cells in the gut, such as macrophages, mast cells, and the brain, such as microglia, astrocytes mediate responses to insults affecting the intestines and CNS.^21^ An example is the overgrowth of gut *Klebsiella* as a strong indicator for infant brain damage (white matter injury) and a marker for enhanced systemic inflammation.^502^
	**Spleen** **Gut** **Immune**	Systemic inflammation induced by traumatic brain injury (TBI) impacts the spleen and gut, and their respective responses can worsen the neuroinflammatory damage caused by the injury. Physical exercise that focuses on modulating the immune system is a potential treatment for postinjury conditions.^110^
	**Sensory** **Vasculature**	Ocular diseases like glaucoma, optic neuropathy, macular degeneration, and diabetic retinopathy are associated with damage to not only the retina and optic nerve but also the brain. Indirect influences can involve intracerebral pressure, eye movements, top-down modulation (attention, cognition), and the release of stress hormones.^503,504^ Residual vision can be rescued by means including vision restoration training, noninvasive brain stimulation, or medications that enhance blood flow.
**Heart**	**Lung** **Immune**	Symptoms of critically ill patients without heart-related conditions manifest as myocardial injury following initial pulmonary damage. Mechanisms involve roles of angiotensin converting enzyme receptor 2 (ACE2), immune dysregulation, hypoxia induced cardiac damage. Another pathology is pulmonary hypertension, for which many review articles have addressed.^505^
	**Spleen** **Immune**	In acute myocardial infarction, the spleen acts as a monocyte reservoir, releasing immune cells to the cardiac injury site with specific spatiotemporal distribution, revealing the mechanism of dynamic interaction among the heart, spleen, and immune system through monocytes to regulate repair.^506^
**Lung**	**Spleen** **Kidney**	Splenic IL-10 serves protective function against kidney injury, lung capillary leak and neutrophil induced lung damage in ischemia‒reperfusion injury.^507^
**Liver**	**Skin** **Immune**	Increased inflammation, characterized by enhanced proinflammatory adipokines and hepatokines, is a common phenomenon identified in psoriasis, which puts patient at risk for MAFLD.^508^
	**Gut** **Kidney** **Immune**	Mild inflammation is the underlying cause for liver–gut–kidney related diseases, leading to fibrosis, dysbiosis, disrupted intestinal integrity, dyslipidemia, and insulin resistance.^509^
	**Gut** **Muscle**	Sarcopenia in patients with cirrhosis is linked to changes in the gut-liver-muscle axis, including a disrupted gut microbiome composition, metabolic pathways and increased inflammatory markers.^510^
	**Gut** **Adipose**	Obesity induced inflammation leads to changes in gut integrity and homeostasis. The release of proinflammatory mediators into portal circulation adversely impacts the liver, driving the development of insulin resistance and liver pathologies.^511^
**Kidney**	**Bone** **Endocrine**	Deficiency in bone morphogenetic proteins (BMPs) in CKD lead to decreased bone turnover, for which the body attempts to rescue through hyperparathyroidism to increase turnover.^512^ In addition, endocrine factors such as FGF23 and αKlotho are implicated in many animal and clinical studies to be relevant for the kidney–bone axis.^513^ Treating hyperparathyroidism is found to be effective in delaying bone remodeling as indicated in animal models.^512^
	**Skin** **Immune**	The cross-talk between the kidney and skin is pronounced in autoimmune conditions, such as systemic lupus erythematosus (SLE). Mediators from the inflamed skin can travel through circulation and reach the kidney, exacerbating distant injury.^514^
	**Spleen** **Immune**	The kidney, spleen, and immune system form a dynamic network via immune cells (e.g., Tregs, macrophages) and cytokines (e.g., IL-2), with the spleen regulating renal repair and immune dysregulation affecting kidney disease progression.^515^
	**Gut** **Muscle**	The combination of symptoms, including compromised renal function, increased level of urea and bacterial species that metabolizes urea, and muscle wasting, is observed both in end-stage renal disease (ESRD) patients and elderly individuals. Reduced microbial metabolites such as SCFAs, acetate, propionate, is the partial cause of such observation. Resuming a high-fiber diet, which presumably fuels gut microbes in producing SCFAs, can potentially amend the kidney–gut–muscle axis in both conditions.^516^
	**Gut** **Immune**	Evidence from both animal and clinical studies have provided robust rationale for the role of gut microbiota homeostasis (its composition, metabolism, integrity) in maintaining renal function.^517^
**Gut**	**Muscle** **Immune**	Excessive training can lead to dysbiosis and drive local inflammatory responses. In addition, the gut–muscle axis may contribute to the development of muscle wasting disorders through mechanisms, including the transmission of pro-anabolic signals from dietary nutrients, modulation of inflammation, and insulin sensitivity as one ages.^518^
	**Skin** **Immune**	The composition of the gut microbiome impacts the signaling mechanisms involved in maintaining epidermal differentiation, thereby influencing the overall homeostasis of the skin. Dietary changes (a shift to low-fat, high-protein, high-fiber diet), the use of antibiotics, pre/probiotics and novel biologic drugs that target inflammatory mediators.^519^
**Adipose**	**Muscle** **Bone** **Neuron**	Irisin, an adipokine, plays a role in various physiological processes: It induces the browning of beige precursor fat cells within white adipose tissue, driving energy expenditure. It affects bone metabolism by promoting osteoblast differentiation and suppressing osteoclast. It also influences hippocampal neurogenesis and neural differentiation in mice. Evidence presents irisin as a mediator between the four components.^520^
**Muscle**	**Eye** **Brain**	Muscle-Eye-Brain (MEB) is a combination of muscular dystrophy, eye and brain anomalies and cognitive disability, including limited communication skills. Suspected genetic factor is the mutation in *B3GALNT2*, a glycosyltransferase.^521^
**Immune**	**Neuro** **Endocrine**	The three systems interact under many pathologic states. We take chronic stress as an example, where increased inflammatory cytokine IL-1β levels are transformed to a nervous signal. The nervous system triggers the release of melatonin and cortisol of the endocrine system utilizes to counterbalance IL-1β. Other examples could be found in these reviews^522,523^

Analysis revealed that the brain–gut, gut-liver, and brain–heart regions were the most studied organ pairs, accounting for 17.31% (8746 articles), 5.94% (3000 articles), and 3.82% (1931 articles) of the retrieved publications, respectively. The organs with the highest research coverage included the brain (17,735 articles, 35.10%), gut (16,169 articles, 32.00%), and liver (13,916 articles, 27.54%), underscoring their central roles in the field. However, cross-talk involving pairs such as stomach-adipose, stomach-bone, and spleen-adipose pairs remains underexplored. This uneven distribution suggests a need for future studies to address neglected organ pairs to fully elucidate the complexity of multiorgan networks.

During the review process, we found that the circulatory, nervous, endocrine, and immune systems play critical roles in mediating interorgan interactions and regulating communication and functional coordination between organs. These systems are involved in nearly all interorgan interactions, forming a complex regulatory network. The nervous system rapidly regulates organ interactions through pathways such as the vagus nerve,^17,18^ whereas the endocrine system transmits information across multiple organs via hormonal signals such as insulin and cortisol.^19,20^ The immune system participates in the dynamic balance between organs through inflammatory responses and immune surveillance,^21–23^ and the circulatory system provides the physical foundation for interorgan communication by delivering nutrients, oxygen, and chemical signals through blood flow.^24–26^ These four systems not only collaboratively mediate interorgan dialog but also offer a crucial research perspective for understanding overall health and disease states.

**Fig. 2:**
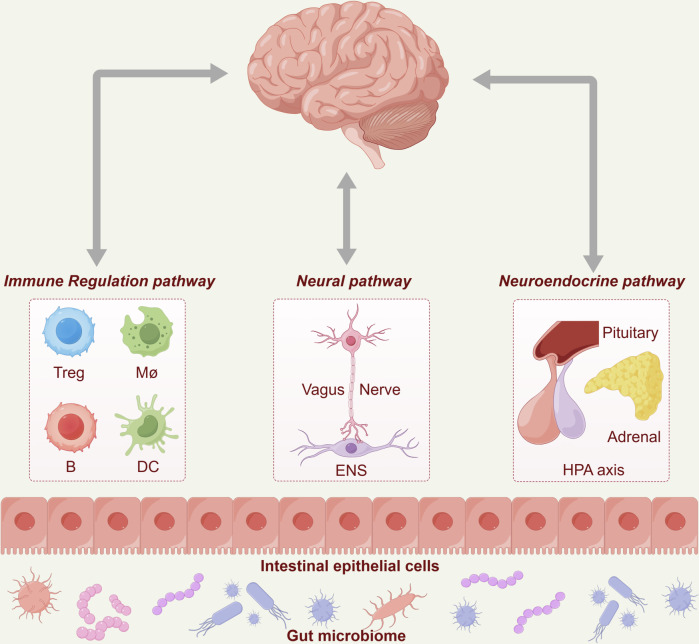
Brain‒gut cross-talk. The diagram illustrates how the brain interacts with the gut microbiome through three primary pathways: the immune regulation pathway, involving regulatory T cells (Tregs), macrophages (Møs), B lymphocytes (B cells), and dendritic cells (DCs); the neuronal pathway, which is mediated by the vagus nerve and the enteric nervous system (ENS); and the neuroendocrine pathway, which is governed by the hypothalamic‒pituitary‒adrenal (HPA) axis, along with associated neurotransmitters, gut hormones, and cytokines. This visualization highlights the intricate mechanisms underlying brain‒gut communication and the critical role of the gut microbiome in this process. The figure was created by Figdraw (www.figdraw.com)

**Fig. 3:**
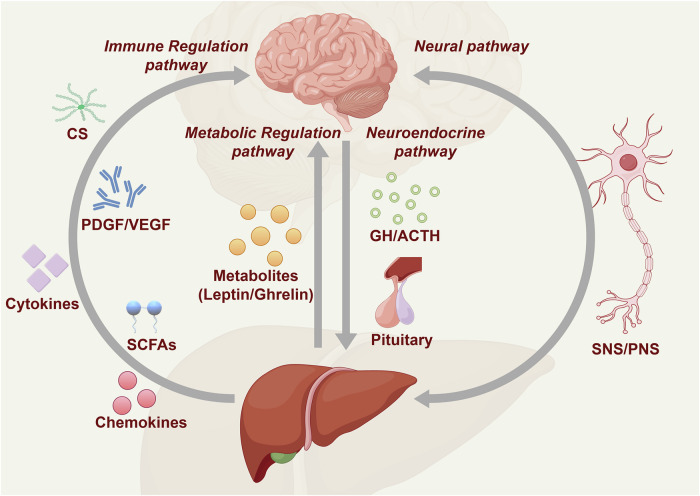
Brain‒liver cross-talk. This diagram illustrates the bidirectional communication between the brain and liver through neural, neuroendocrine, and immune/metabolic pathways. The vagus nerve acts as the primary neural conduit, transmitting signals between the brain and liver and influencing liver functions such as metabolism and inflammation regulation. The pituitary gland, through the release of growth hormone (GH) and adrenocorticotropic hormone (ACTH), plays a critical role in the neuroendocrine pathway, impacting liver function and systemic metabolism. Conversely, the liver produces cytokines, enzymes, and metabolites, including platelet-derived growth factor (PDGF), vascular endothelial growth factor (VEGF), and corticosteroids (CSs), which affect the brain via immune and metabolic pathways. SCFAs and other metabolites, such as leptin and ghrelin, further contribute to metabolic regulation. The SNS and PNS are key components of the neural pathways that mediate this communication. This complex network of interactions highlights the significant role of brain‒liver communication in maintaining systemic health and homeostasis

**Fig. 4:**
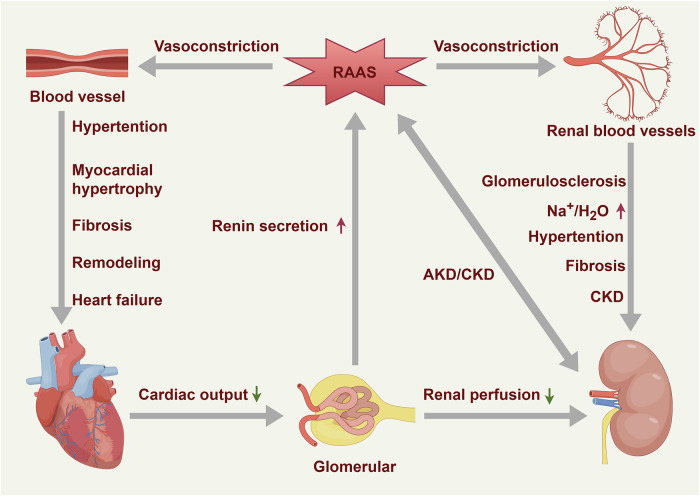
Heart–kidney cross-talk. The diagram illustrates the bidirectional communication between the heart and the kidneys through the renin–angiotensin–aldosterone system (RAAS). The activation of the RAAS is triggered by reduced renal perfusion, leading to increased renin secretion. RAAS activation causes vasoconstriction, resulting in elevated blood pressure and a series of pathological changes, including myocardial hypertrophy, fibrosis, cardiac remodeling, and HF. Additionally, the RAAS leads to sodium (Na⁺) and water (H₂O) retention, increasing blood volume and pressure, which in turn causes renal fibrosis and glomerulosclerosis, leading to acute kidney disease (AKD) and chronic kidney disease (CKD). These renal issues further decrease renal perfusion, continuously activating the RAAS and creating a vicious cycle, worsening both cardiac and renal function. This diagram highlights the critical role of the RAAS in regulating the pathological interactions between the heart and kidneys, highlighting its significant contribution to the progression of cardiovascular and renal diseases

**Fig. 5:**
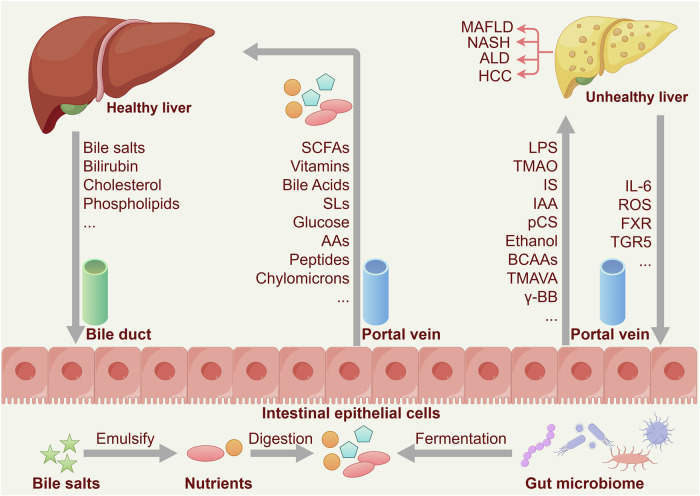
Gut‒liver cross-talk. In a healthy liver, bile salts, bilirubin, cholesterol, and phospholipids are secreted via the bile duct, facilitating fat emulsification, digestion, and nutrient absorption in the intestine. The gut microbiota ferments nutrients to produce beneficial metabolites such as short-chain fatty acids (SCFAs), vitamins, secondary bile acids (SBAs), glucose, amino acids (AAs), peptides, and chylomicrons, which are transported back to the liver via the portal vein, forming a supportive cycle for liver health and systemic homeostasis. In contrast, an unhealthy liver, associated with metabolic-associated fatty liver disease (MAFLD), nonalcoholic steatohepatitis (NASH), alcoholic liver disease (ALD), or hepatocellular carcinoma (HCC), is linked to gut microbiota dysbiosis, leading to the production of harmful metabolites such as lipopolysaccharides (LPS), trimethylamine N-oxide (TMAO), and indoxyl sulfate (IS). These toxins enter the liver via the portal vein, exacerbating damage and inflammation. A damaged liver releases interleukin (IL)-6 and reactive oxygen species (ROS), increasing gut permeability and amplifying inflammation while impairing the feedback loops mediated by farnesoid X receptor (FXR) and G protein-coupled bile acid receptor 1 (TGR5). FXR typically enhances barrier integrity and glucagon-like peptide-1 (GLP-1) secretion, whereas TGR5 suppresses inflammation via cAMP signaling; their dysfunction indirectly affects gut-related functions

**Fig. 6:**
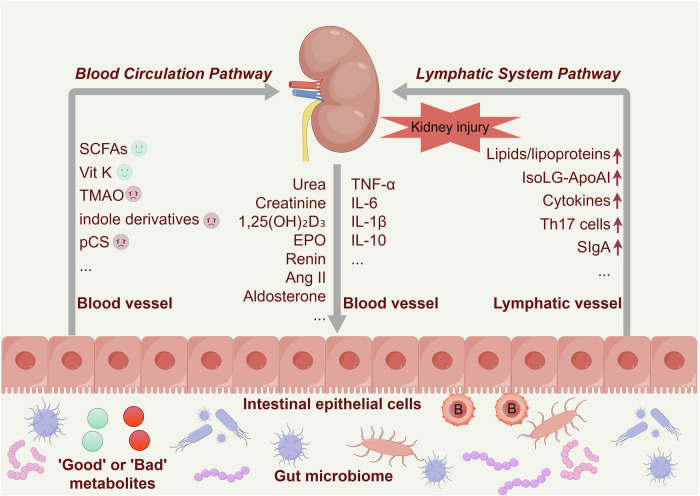
Gut–kidney cross-talk. This diagram illustrates the bidirectional relationship of the gut–kidney axis, highlighting how metabolites produced by the gut microbiome affect kidney function via the bloodstream and how substances secreted by the kidneys (such as renin, erythropoietin (EPO), and toxic metabolites) influence the gut and its immune cells through blood circulation. The “good” metabolites include SCFAs and vitamin K (Vit K), whereas the “bad” metabolites include p-cresyl sulfate (pCS), indole derivatives (such as indole-3-acetic acid), indoxyl sulfate (IS), trimethylamine N-oxide (TMAO), and lipopolysaccharide (LPS). Kidney dysfunction or injury leads to elevated levels of tumor necrosis factor-alpha (TNF-α), interleukin-6 (IL-6), interleukin-1 beta (IL-1β), and interleukin-10 (IL-10), alongside increased secretion of lipids/lipoproteins, isolevuglandin-modified apolipoprotein A-I (IsoLG-ApoAI), cytokines, T-helper 17 cells (Th17 cells), and secretory immunoglobulin A (SIgA). These factors, in turn, influence the gut microbiota composition and activity, exacerbating the progression of kidney disease

The subsequent subsections delve into organ cross-talk centered on the brain, heart, and gut, with mechanistic diagrams (Figs. 2–6) illustrating key signaling pathways. Additionally, other cross-talk, axes, and network interactions are systematically compiled in Tables 1 and 2 for reference and comparison. These analyses aim to provide a framework for researchers, fostering deeper exploration of disease-related organ cross-talk and the development of therapeutic strategies.

### Brain

The brain serves as the central command hub of human physiology and behavior, orchestrating a vast array of functions that define our existence—from the coordination of movement and processing of sensory input to the regulation of emotions, cognition, and higher-order decision-making. This remarkable organ continuously integrates and interprets information from both the internal milieu and the external environment, ensuring adaptive responses that maintain survival and well-being.^27–30^ Beyond its canonical roles in neural processing, the brain engages in intricate bidirectional communication with peripheral organs, including the gut, liver, heart, adipose tissue, and beyond, forming a complex network of interorgan cross-talk that underpins systemic homeostasis.^31–33^ These interactions are mediated through an array of signaling pathways—neural, hormonal, immune, and metabolic—that collectively enable the brain to exert top-down control while simultaneously receiving feedback from peripheral systems, creating a dynamic interplay critical to health.

Brain-centered cross-talk is not merely a passive exchange but rather a sophisticated dialog that shapes physiological states and influences disease trajectories. Advances in neuroscience and systems biology have illuminated how disruptions in these communication networks contribute to a spectrum of pathologies, ranging from neuropsychiatric disorders to metabolic syndromes and cardiovascular diseases.^32,34^ For example, the regulatory influence of the brain over peripheral organs can modulate metabolic processes, immune responses, and even tissue repair, whereas signals from these organs—such as microbial metabolites from the gut or inflammatory cytokines from the liver—can, in turn, alter brain function, behavior, and resilience to stress.^35^ Interestingly, the blood‒brain barrier (BBB) modulates these interactions by filtering which peripheral signals reach the brain, and its impairment—due to inflammation, oxidative stress, or aging—can amplify disease progression by allowing harmful substances to infiltrate the central nervous system.^36^ Research into these brain‒organ axes has revealed their profound therapeutic potential, offering novel insights into the pathogenesis of multiple diseases and paving the way for innovative treatment strategies that target these interconnected systems.

The following subsections delve into specific examples of brain-centered cross-talk—namely, with the gut, liver, and heart—and explore the molecular mechanisms, signaling pathways, and biological implications of these relationships. By highlighting the results from cutting-edge studies, we aim to highlight the pivotal role of the brain as a conductor and recipient within the organ network, underscoring its importance in maintaining physiological balance and vulnerability to dysregulation in disease states. These explorations not only deepen our understanding of human biology but also set the stage for precision medicine approaches that leverage brain‒organ interactions to improve health outcomes.

#### Brain‒gut cross-talk

##### Mechanisms of brain–gut cross-talk

Brain‒gut cross-talk refers to the complex interactions between the brain and various components of the gastrointestinal tract. This relationship is founded on three pathways, namely, the neural, neuroendocrine, and immune pathways, which collectively form the basis of the gut‒brain interaction^31,37–39^ (Fig. 2). This section focuses on the specific mechanisms proposed for brain‒gut cross-talk.

Neural communication hinges on the vagus nerve, which transmits signals bidirectionally between the gut and brain.^40^ Gut distension stimulates the vagus nerve, causing the release of acetylcholine or catecholamines, which interact with enteroendocrine cells. This interaction results in the production of neuropeptides, gut hormones, neurotransmitters, and microbe-associated molecular patterns (MAMPs).^41,42^ Additionally, compounds derived from microbes, such as short-chain fatty acids (SCFAs), secondary bile acids (2BAs), and tryptophan metabolites, facilitate communication between the gut and the central nervous system (CNS).^43^ Some of these compounds can stimulate immune cells to release cytokines or directly interact with the vagus nerve, thereby increasing signaling. Furthermore, certain compounds can cross the intestinal barrier, enter the bloodstream, and potentially cross the blood‒brain barrier, affecting brain function.^44,45^ Interestingly, research has shown that the dorsal motor nucleus of the vagus nerve (DMV) also plays a role in regulating fat absorption, where DMV inhibition reduces fat absorption and leads to weight loss, whereas its activation has the opposite effect. The natural compound puerarin can also reduce fat absorption by suppressing the DMV-vagus pathway.^3^

The neuroendocrine pathway, especially the hypothalamic‒pituitary‒adrenal (HPA) axis, plays a crucial role in gut‒brain cross-talk.^46–48^ The HPA axis is responsible for a range of daily functions, such as digestion, immunity, and stress response.^49^ Exposure to external stimuli, such as stress or depression, triggers neurotransmitter-mediated signal transmission. This process activates the hypothalamic paraventricular nucleus to release corticotropin-releasing hormone (CRH), which stimulates the anterior pituitary gland to produce and release adrenocorticotropic hormone (ACTH).^50^ ACTH prompts the adrenal cortex to synthesize glucocorticoids, mainly cortisol. Cortisol impairs gastrointestinal motility and compromises the integrity of intestinal epithelial cells, resulting in symptoms such as diarrhea.^31^ Additionally, it can trigger a series of immune responses in the gastrointestinal tract. Proinflammatory cytokines, such as TNF-α, IFN-γ, and IL-6, further weaken the suppressive mechanism of the HPA axis, leading to its ongoing activation and creating a feedback loop. These cytokines can also potentially damage the brain, either through the vagus nerve or directly. These processes often involve the downregulation of serotonin pathways and brain-derived neurotrophic factors, which are linked to emotional and psychological feedback. Therefore, long-term stress may predispose certain individuals to inflammatory bowel disease (IBD), whereas chronic intestinal inflammation can increase the risk of depression.^51–53^

The immune system also plays a role in gut‒brain cross-talk. The innate immunological inflammasome pathway serves as a conduit for communication between microorganisms and the CNS. Dysregulation of these pathways may lead to excessive activation of the brain inflammasome. The intestinal microbiota continuously stimulates inflammasomes in the intestine, indirectly influencing the brain.^54–56^ The activation of inflammasomes is associated with neuroinflammatory disorders in the CNS, such as multiple sclerosis (MS), Alzheimer’s disease (AD), postpartum depression (PPD), and neuropsychiatric symptoms (NPS).^57–62^ Inflammatory pathways might also play a role in the development of depressive behaviors. Neurological conditions such as cognitive decline, autism spectrum disorder (ASD), and obesity-related neurobehavioral symptoms often coincide with intestinal inflammation and proinflammatory serological profiles, which are linked to cognitive and behavioral impairments.^63–67^

##### From dysfunction to intervention

The relationship between ASD and the microbiome suggests a fascinating bidirectional causality: microbial dysbiosis may influence brain development through immune, metabolic and neurotransmitter pathways, potentially contributing to ASD, whereas intriguingly, the behavioral and dietary habits of individuals with ASD may also alter the gut environment, leading to microbial imbalance.^68^ Similarly, the link between PD and the brain–gut–microbiome axis is hotly debated: the gut–first hypothesis proposes that gut microbial dysbiosis may trigger neurodegeneration via the vagus nerve or immune system, which is quite compelling, whereas interestingly, the brain–first hypothesis suggests that brain pathology (e.g., α-synuclein aggregation) may occur first and affect the gut microbiota.^69,70^ Notably, both mechanisms may interact, jointly driving disease progression in a complex, intertwined dance. Notably, dysfunction of the brain‒gut axis is also associated with gastrointestinal disorders (e.g., IBD, irritable bowel syndrome [IBS]) and neuropsychiatric conditions (e.g., anxiety, depression, neurodegeneration), further highlighting the profound interconnectedness of these systems.^70–73^

Therapeutic strategies targeting this axis are multifaceted. Probiotics and prebiotics restore microbial balance, increasing SCFA production to increase gut barrier function and reduce inflammation, with clinical evidence supporting their efficacy in alleviating IBS and depression symptoms.^74^ Pharmacological interventions, such as selective serotonin reuptake inhibitors (SSRIs), modulate both brain serotonin and gut motility, whereas vagus nerve stimulation (VNS) reduces inflammation and improves psychiatric outcomes.^75^ Dietary approaches, such as the Mediterranean diet, promote microbial health and systemic anti-inflammatory effects, and fecal microbiota transplantation (FMT) shows promise for IBD and neuropsychiatric disorders by recalibrating the gut microbial composition.^76,77^

In conclusion, brain‒gut cross-talk represents a sophisticated and dynamic communication network that integrates neural, neuroendocrine, and immune pathways to maintain homeostasis between the gastrointestinal tract and the central nervous system. Dysregulation of this axis underlies a wide range of disorders, from gastrointestinal conditions such as IBD and IBS to neuropsychiatric diseases such as depression and neurodegeneration. Emerging therapeutic strategies, including probiotics, pharmacological interventions, dietary modifications, and neuromodulation techniques, offer promising avenues for restoring brain‒gut axis balance and improving patient outcomes. As research continues to unravel the intricate mechanisms of this bidirectional communication, it becomes increasingly clear that targeting the brain‒gut axis holds significant potential for advancing the treatment of both gastrointestinal and neuropsychiatric disorders, ultimately bridging the gap between physical and mental health.

#### Brain‒liver cross-talk

##### Mechanisms of brain‒liver cross-talk

The brain‒liver axis plays a crucial role in regulating brain function and behavior, particularly in metabolism and inflammatory responses.^16,78–82^ As a key metabolic organ, the liver is responsible for regulating glucose and lipid metabolism and detoxifying harmful substances. Additionally, the liver produces signaling molecules, such as bile acids (BAs), which can influence brain function and behavior through the circulatory system.^83–85^

Bidirectional communication within this axis involves neural, neuroendocrine, and metabolic pathways (Fig. 3). The brain‒liver axis mediates cross-talk through the sympathetic nervous system (SNS) and parasympathetic nervous system (PNS). The SNS, via splanchnic nerves, releases norepinephrine to stimulate hepatic adrenergic receptors, promoting glycogenolysis, gluconeogenesis, and inflammation.^80^ Conversely, the PNS, through the vagus nerve, releases acetylcholine to activate muscarinic receptors, inhibiting glucose production, increasing glycogenesis, and relaying metabolic signals to the brain to modulate appetite and cognition.^79,86^ This SNS-PNS interplay dynamically balances metabolism and immune responses within the axis, supporting homeostasis. The brain modulates liver function through neurotransmitters (e.g., norepinephrine, serotonin) and neuroendocrine signals (e.g., corticotropin-releasing hormone, CRH), which are transmitted via the vagus nerve or bloodstream to hepatic receptors.^75,87–90^ This process modulates liver metabolism, immune responses, and inflammation. For example, during fasting, the activation of hypothalamic AgRP neurons leads to increased corticosterone secretion, which increases hepatic autophagy and metabolic remodeling.^91^ Moreover, growth hormone (GH) and ACTH released from the pituitary gland are also key players in the brain‒liver axis. These hormones affect liver metabolism, especially under stress.^92–94^

In addition to its influence on the brain, the liver can also affect brain function by releasing metabolites and hormones. For example, the liver synthesizes cholesterol and produces BAs, which can travel to the brain through the circulatory system. These molecules play a role in neurodevelopment, memory, and cognitive functions. BAs can influence brain function and behavior by regulating brain–gut communication and activating receptors within the brain.^85^ Furthermore, BAs improve metabolism and have antiobesity effects by activating Takeda G protein-coupled receptor 5 (TGR5) in peripheral tissues.^95^ Other metabolites produced by the liver, such as lysophosphatidylcholine (LPC), are quickly converted to lysophosphatidic acid (LPA) in the bloodstream, which then alters brain neuronal activity and appetite regulation.^96,97^

Additionally, signaling factors such as platelet-derived growth factor (PDGF), vascular endothelial growth factor (VEGF), and cytokines, as well as other metabolites, circulate to the brain and modulate neural activity.^98,99^ The activity of hepatic soluble epoxide hydrolase (sEH) influences cerebral Aβ metabolism and AD pathogenesis.^32,100,101^ Modifying the liver–brain interaction in response to environmental stimuli holds potential as a therapy to prevent AD. Suppressing the hepatic Ephx2 gene, which encodes sEH, has been shown to reduce neurological deficits caused by traumatic brain injury and increase neurological function recovery.^100^ Similarly, ketone bodies produced in the liver have been found to enhance cognitive function and mood in both animal models and humans.^102–104^

After exercise, plasma levels of glycosylphosphatidylinositol (GPI)-specific phospholipase D1 (Gpld1), a liver-derived enzyme, increase, with higher Gpld1 levels associated with improved cognitive function in aged mice. Elevating systemic Gpld1 concentrations in aged mice improves age-related regenerative and cognitive impairments.^105^ Moreover, the liver significantly influences feeding behavior in mice. During fasting, the liver secretes LPC, which is quickly converted to LPA in the bloodstream, increasing LPA levels and altering brain neuronal activity to increase the organism’s appetite.^106^

##### From dysfunction to targeted interventions

Dysregulation of the brain‒liver axis underlies multiple diseases. Hepatic encephalopathy, a complication of cirrhosis, arises from neurotoxin accumulation (e.g., ammonia), impairing brain function.^78,107^ Neurodegenerative disorders such as AD and Parkinson’s disease (PD) correlate with altered liver metabolism, including dysregulated BA and cholesterol homeostasis.^108,109^ Metabolic disorders, such as obesity and type 2 diabetes, often involve disrupted brain–liver communication, exacerbating both hepatic and neurological symptoms.^83,84,95^

Therapeutic strategies targeting the brain‒liver axis hold promise. TGR5 agonists improve metabolism and cognition by modulating BA signaling,^95^ whereas sEH inhibitors reduce neuroinflammation and increase TBI recovery.^110^ The ketogenic diet, by boosting hepatic ketone production, ameliorates neurodegenerative symptoms.^104,111^ Gene therapies, such as the downregulation of hepatic Ephx2 (which encodes soluble epoxide hydrolase (sEH)), ameliorate TBI-induced neurological deficits and promote recovery of neurological function.^100^ In an immune-mediated liver‒brain communication pathway, peripheral CCR2(+) monocytes migrate into the brain parenchyma in response to activated microglia expressing MCP-1/CCL2; inhibiting monocyte infiltration significantly improves pathological behaviors linked to liver inflammation.^89^ Additionally, brain‒liver axis therapies include epigenetic regulation and approaches related to β-amyloid (Aβ) metabolism.^13,112,113^

In summary, the brain‒liver axis orchestrates a complex interplay of neural, neuroendocrine, metabolic, and immune pathways that regulate metabolism, inflammation, and behavior, with profound implications for both physiological homeostasis and disease states. Dysregulation of this axis contributes to conditions such as hepatic encephalopathy, neurodegenerative disorders, and metabolic diseases, highlighting the critical role of the liver in brain health and vice versa. Emerging therapeutic strategies—ranging from TGR5 agonists and sEH inhibitors to ketogenic diets, gene therapies targeting Ephx2, immune modulation, and epigenetic approaches—demonstrate the potential to restore brain‒liver communication, offering innovative avenues for managing related pathologies and advancing personalized medicine.

#### Brain‒heart cross-talk

##### Mechanisms of brain‒heart cross-talk

The brain‒heart axis constitutes a critical bidirectional network orchestrating cardiovascular and cerebrovascular homeostasis, which is mediated by neuroendocrine, neuroimmune, and hemodynamic pathways. The brain governs cardiac function through the central autonomic nervous system (ANS), integrating sensory inputs and modulating output via neural circuits, whereas the heart influences cerebral function through blood flow dynamics and hormonal signaling.^114^ This intricate interplay, pivotal to physiological equilibrium, is profoundly disrupted in disease states, driving a spectrum of cardiovascular and neurological pathologies.^5,16,49,103,115–119^

The ANS, encompassing the SNS and PNS, serves as the primary neural conduit. SNS activation releases norepinephrine (NE) onto cardiac β-adrenergic receptors, accelerating heart rate, increasing atrioventricular conduction, and amplifying myocardial contractility — an adaptive response to stressors such as exercise or hemorrhage.^120^ Conversely, the PNS, via vagal efferents, releases acetylcholine onto muscarinic receptors, slowing heart rate and reducing contractility, as seen during rest or digestion.^121^ This balance is tightly regulated by brainstem nuclei (e.g., the nucleus tractus solitarius and dorsal motor nucleus) and hypothalamic circuits, integrating baroreceptor and chemoreceptor feedback from the carotid sinus and aortic arch. In pathological contexts—such as chronic stress or hypertension—SNS overactivation and PNS suppression skew this equilibrium, elevating catecholamine levels and triggering tachycardia, arrhythmias, or heart failure (HF).^117,122–124^ Excessive SNS drive also stimulates juxtaglomerular cells to release renin, activating the renin–angiotensin system (RAS), which induces vasoconstriction, promotes myocyte hypertrophy, and fosters inflammation, exacerbating atherosclerotic cardiovascular disease (ASCVD) and HF progression.^125–127^

Neuroendocrine pathways further amplify brain‒heart cross-talk. Under stress, the hypothalamic‒pituitary‒adrenal (HPA) axis triggers corticotropin-releasing hormone (CRH) release, promoting pituitary adrenocorticotropic hormone (ACTH) secretion and adrenal cortisol production. Chronic cortisol elevation induces water‒sodium retention, hypertension, and dyslipidemia, straining cardiac workload and contributing to mood disorders such as depression and anxiety—conditions that are often comorbid with HF.^128^ The sympathetic-adrenal-medullary (SAM) system, intertwined with the SNS, releases epinephrine and NE from the adrenal medulla, acutely increasing the heart rate and contractility during fight-or-flight responses. However, prolonged SAM activation fosters oxidative stress and endothelial dysfunction, accelerating atherosclerosis. These neuroendocrine cascades, while adaptive short term, become maladaptive in chronic states, linking psychological stressors to cardiovascular risk.^129^

The neuroimmune axis connects the brain and heart via inflammatory signaling. SNS overstimulation activates lymphoid tissues (e.g., the spleen and bone marrow), releasing NEs to mobilize monocytes and neutrophils, which produce proinflammatory cytokines (e.g., IL-6 and TNF-α). These cytokines, via the cholinergic anti-inflammatory pathway, exacerbate myocardial inflammation and fibrosis, as seen in postmyocardial infarction (MI) remodeling.^130^ Brain injuries, such as stroke, further amplify this response, releasing damage-associated molecular patterns (DAMPs) that trigger systemic inflammation, impairing cardiac function.^131^ Conversely, cardiac stress signals back to the brain, with cytokines crossing the BBB to activate microglia, perpetuating neuroinflammation and cognitive decline.^117^

The heart reciprocally influences the brain through hemodynamic and hormonal mechanisms. In HF, reduced cardiac output causes cerebral hypoperfusion, impairing neuronal metabolism and manifesting as cognitive deficits or mood disturbances—termed cardiogenic dementia.^132^ Cardiac hormones, such as atrial natriuretic peptide (ANP) and B-type natriuretic peptide (BNP), cross the BBB to regulate hypothalamic circuits, modulating emotional processing and fluid balance.^133–135^ Inflammation and oxidative stress amplify this feedback; RAS overactivation elevates reactive oxygen species (ROS), damaging the vascular endothelium and neurons and linking ASCVD and HF to neuroinflammatory disorders such as depression.^125,136^ Post-MI, the myocardial release of IL-1β and matrix metalloproteinases further disrupts the cerebral vasculature, increasing stroke risk.^137,138^

##### From dysfunction to intervention

Brain‒heart cross-talk drives diverse pathologies. In MI, brain-mediated SNS overdrive and HPA activation exacerbate infarct size and arrhythmogenesis,^139^ whereas poststroke cardiac dysfunction (e.g., Takotsubo cardiomyopathy) reflects ANS dysregulation, with catecholamine surges weakening myocardial contractility.^115,140^ Hypertension, a bidirectional outcome, stems from RAS and SNS hyperactivity, with cerebral hypoperfusion fostering white matter lesions.^141–143^ Intermittent hypoxia in sleep apnea triggers SNS surges and RAS activation, promoting HF and atrial fibrillation.^144,145^ Neurodegenerative diseases such as Alzheimer’s disease correlate with microvascular dysfunction, potentially worsened by cardiac insufficiency, underscoring shared vascular aetiologies.^146^

Therapeutic strategies target this cross-talk’s multifaceted nature. VNS restores ANS balance, reducing inflammation and improving HF outcomes by increasing parasympathetic tone, as validated in clinical trials.^123^ RAS inhibitors (e.g., ACE inhibitors and ARBs) lower blood pressure, mitigate myocardial remodeling, and protect the cerebral vasculature, which are dual benefits seen in hypertension and poststroke care.^147^ Beta-blockers attenuate SNS overdrive, easing cardiac strain and arrhythmia risk in HF and MI.^148^ Emerging therapies, such as SGLT2 inhibitors, reduce cardiac preload and neuroinflammation, offering potential in HF and diabetes-related brain injury.^149,150^ Lifestyle interventions—exercise and stress reduction—bolster ANS resilience, mitigating cross-talk disruptions.^151^

In summary, brain‒heart cross-talk represents a dynamic and intricate interplay of neural, endocrine, immune, and hemodynamic pathways that maintains cardiovascular and cerebral homeostasis. Dysregulation of this axis underpins a wide array of pathologies, from myocardial infarction and heart failure to stroke, hypertension, and neurodegenerative disorders, driven by mechanisms such as sympathetic overactivation, inflammatory cascades, and hormonal imbalances. Therapeutic interventions, including vagus nerve stimulation, RAS inhibitors, beta-blockers, and emerging treatments such as SGLT2 inhibitors, alongside lifestyle modifications, offer promising avenues to restore balance and mitigate disease progression. Understanding and targeting this bidirectional network not only illuminates the shared etiology of cardiovascular and neurological conditions but also paves the way for integrated, cross-talk-informed approaches to improve patient outcomes across these intertwined domains.

### Heart

The heart, a vital engine of cardiovascular homeostasis, drives systemic circulation through specialized cells—cardiomyocytes, pacemaker cells, and vascular networks—to deliver oxygen, nutrients, and signals while clearing waste. In addition to its role as a pump, it engages in dynamic, bidirectional cross-talk with multiple organs via neural, hormonal, immune, and metabolic pathways.^5,26,152–155^ This interplay sustains physiological balance and underscores the heart’s central role in a multiorgan network essential to health.

This cross-talk is a finely tuned orchestration in which the heart influences and is shaped by the functional states of its partner organs. For example, through the autonomic nervous system and endocrine signals such as natriuretic peptides, the heart modulates renal filtration, pulmonary gas exchange, and hepatic metabolism, while receiving reciprocal feedback—such as inflammatory mediators from the gut or pressure signals from the lungs—that can either bolster its performance or precipitate dysfunction.^156,157^ In states of health, these interactions ensure efficient resource allocation and adaptive responses to stressors such as exercise or injury. However, in pathological conditions such as HF, this delicate balance falters, triggering a cascade of disruptions that ripple across organ systems. Reduced cardiac output in HF, for example, can impair renal perfusion, exacerbate pulmonary congestion, and strain hepatic function, whereas organ-specific maladies—such as gut dysbiosis or liver dysregulation—can, in turn, intensify cardiac stress, fueling a vicious cycle of multiorgan decline.^10,158–161^

The importance of these heart-centric interactions is increasingly evident in clinical and research domains, where understanding their molecular underpinnings offers insights into disease mechanisms and therapeutic opportunities. Advances in imaging, omics technologies, and computational modeling have revealed how perturbations in one organ reverberate through this network, amplifying dysfunction or resilience depending on the context. The following subsections explore the cross-talk between the heart and the kidneys, lungs, or liver in greater detail, dissecting the hemodynamic, neurohumoral, and inflammatory pathways that define these relationships. By illuminating the role of the heart as both a driver and responder within this multiorgan framework, we aim to underscore its centrality in systemic physiology and highlight the need for integrated therapeutic strategies that address the interconnected nature of cardiovascular health and disease.

#### Heart–kidney cross-talk

##### Mechanisms of heart–kidney cross-talk

The heart‒kidney axis, often termed cardiorenal cross-talk, governs hemodynamic stability and systemic homeostasis through intricate bidirectional interactions. The heart sustains renal perfusion via cardiac output, delivering the oxygen and nutrients essential for filtration, whereas the kidneys regulate blood pressure and extracellular fluid volume through hormonal cascades and waste clearance.^162–165^ This delicate balance, critical for physiological coordination, collapses in cardiorenal syndrome—a hallmark of HF—where dysfunction of the heart precipitates pathology in the kidney, amplifying systemic stress^166,167^ (Fig. 4).

##### From dysfunction to targeted interventions

Cardiac impairment profoundly disrupts renal function. In HF, reduced cardiac output lowers renal blood flow, triggering compensatory activation of the renin–angiotensin–aldosterone system (RAAS).^168^ Renin, which is secreted by juxtaglomerular cells that sense hypoperfusion, catalyzes angiotensin II synthesis, inducing afferent arteriolar vasoconstriction, increasing systemic blood pressure, and promoting sodium–water retention via aldosterone.^169^ Initially, adaptive, chronic RAAS overactivation drives myocardial hypertrophy, interstitial fibrosis, and ventricular remodeling, exacerbating HF. Concurrently, elevated renal venous pressure—stemming from right ventricular dysfunction or tricuspid regurgitation—and angiotensin II-mediated efferent arteriolar constriction reduce the glomerular filtration rate (GFR), fostering glomerulosclerosis and tubular atrophy, which are hallmarks of acute kidney disease (AKD) and chronic kidney disease (CKD).^170–172^ Inflammation and oxidative stress amplify this cascade; the ischemic myocardium releases proinflammatory cytokines (e.g., IL-6 and TNF-α) and ROS, damaging renal tubular epithelial cells and the endothelium. Mitochondrial dysfunction in hypoxic tubules further exacerbates injury, perpetuating a vicious cycle of cardiorenal decline.^173^

Conversely, renal dysfunction imposes significant cardiac burdens. In CKD, uremic toxins (e.g., urea, creatinine, indoxyl sulfate, and lipids) accumulate, inducing systemic inflammation and oxidative stress that trigger left ventricular hypertrophy (LVH), diastolic dysfunction, and arrhythmias.^174,175^ Electrolyte imbalances, notably hyperkalemia, disrupt cardiac electrophysiology, increasing ventricular ectopy risk. Acute kidney injury (AKI), often caused by ischemia–reperfusion, results in the release of IL-1β and neutrophil-derived proteases into the circulation, directly impairing cardiomyocyte contractility and increasing arrhythmia susceptibility.^176^ The systemic inflammatory surge from AKI, marked by C-reactive protein (CRP) and reactive oxygen species (ROS), accelerates coronary atherosclerosis, amplifying myocardial ischemia risk.^126,177,178^ This interplay culminates in multiorgan dysfunction syndrome (MODS) in severe cases, with renal hypoxia driving erythropoietin (EPO) deficiency, further taxing cardiac oxygen demand via anaemia.^179^

Additionally, the heart‒kidney axis is underscored in various systemic diseases. Hypertension reflects chronic RAAS and SNS hyperactivity, with endothelial dysfunction fostering both cardiac stiffness and renal microangiopathy.^180^ Diabetes exacerbates cross-talk via hyperglycemia-induced glycation, promoting diabetic cardiomyopathy—characterized by diastolic dysfunction—and nephropathy with proteinuria.^181,182^ Sepsis, a hyperinflammatory state, triggers simultaneous heart and kidney failure, with gut-derived lipopolysaccharides (LPS) amplifying cytokine storms (e.g., IL-18 and IFN-γ), impairing myocardial and renal microcirculation.^183,184^ Cardiorenal syndrome type 1 (acute HF causing AKI) and type 2 (chronic HF inducing CKD) exemplify acute versus chronic manifestations, respectively, highlighting temporal dynamics.^162,166,167^

Therapeutic strategies are targeted toward the heart‒kidney feedback loop. RAAS inhibitors—angiotensin-converting enzyme inhibitors (ACEis) such as enalapril and angiotensin receptor blockers (ARBs) such as losartan—reduce preload, alleviate cardiac remodeling, and slow renal fibrosis, cornerstone therapies in HF and AKI/CKD management.^185–187^ Anti-inflammatory agents, such as IL-1β inhibitors (e.g., anakinra), counteract cytokine-driven injury, preserving glomerular and myocardial integrity in AKI and HF.^188–190^ Antioxidants such as N-acetylcysteine (NAC) neutralize ROS, mitigating endothelial oxidative stress and tubular necrosis, particularly AKI/CKD.^191^ SNS modulators, including beta-blockers (e.g., carvedilol), restore autonomic balance, lowering myocardial oxygen demand and increasing renal perfusion in chronic states.^169^ Emerging precision therapies target specific pathways: SGLT2 inhibitors (e.g., dapagliflozin) reduce glomerular hyperfiltration and cardiac preloading, offering dual protection in diabetic cardiorenal disease, whereas nanoparticle-based delivery of antifibrotic agents (e.g., those targeting TGF-β) shows preclinical promise.^150,192–194^ Lifestyle interventions, such as sodium restriction and exercise, bolster hemodynamic stability, complementing pharmacological approaches.^172,195–198^

The heart‒kidney axis exemplifies a tightly interwoven system where cardiac vigor and renal filtration mutually reinforce systemic health. Dissecting its molecular and physiological intricacies—from RAAS-driven fibrosis to cytokine cascades—illuminates shared vulnerabilities and resilience, forging holistic strategies to combat cardiorenal syndromes and their multisystem sequelae.

#### Heart‒lung cross-talk

##### Mechanisms of heart–lung cross-talk

The heart‒lung axis is foundational to systemic oxygenation and circulation, uniting cardiac pumping with pulmonary gas exchange through intricate bidirectional interactions. The heart propels deoxygenated blood via the pulmonary artery to the lungs, where alveolar‒capillary gas exchanges oxygen, returning oxygen-rich blood to the left atrium for distribution. This tightly synchronized process, which is disrupted in cardiopulmonary diseases, exemplifies dynamic cross-talk mediated by hemodynamic, neurohumoral, and inflammatory pathways and is critical for maintaining respiratory and cardiovascular homeostasis.^199–201^

##### From dysfunction to targeted interventions

Cardiac function profoundly shapes lung physiology. In HF, diminished left ventricular (LV) output elevates pulmonary venous pressure, driving interstitial fluid leakage and pulmonary edema, which impairs oxygen diffusion and precipitates dyspnea. This effect is stark in HF with preserved ejection fraction (HFpEF), where stiff ventricles increase pressures during exertion, causing ventilation–perfusion (V–Q) mismatch and reduced exercise tolerance, often exacerbated by pulmonary arteriolar remodeling.^202,203^ Right ventricular (RV) dysfunction, which is prevalent in pulmonary hypertension (PH), heightens RV afterload due to elevated pulmonary vascular resistance (PVR), progressing to hypertrophy and failure—termed cor pulmonale.^204–206^ Chronic HF alters lung mechanics; positive pressure ventilation reduces venous return, compromising cardiac output and worsening V/Q mismatch, particularly in hypovolemic states. Inflammation amplifies this interplay; MI or HF release proinflammatory cytokines (e.g., IL-6 and TNF-α), triggering pulmonary endothelial injury, macrophage infiltration, and vascular remodeling, as observed in acute respiratory distress syndrome (ARDS) after cardiac insult.^207,208^ Oxidative stress from the ischemic myocardium generates ROS, further damaging alveolar‒capillary barriers and amplifying hypoxic pulmonary vasoconstriction.^209^

Lung pathology reciprocally burdens the heart. Chronic obstructive pulmonary disease (COPD) induces dynamic hyperinflation, increases intrathoracic pressure (ITP) and PVR, compresses the LV and reduces stroke volume via direct ventricular interaction (DVI), a mechanical constraint worsened by the loss of elastic recoil in emphysema. This also strains the RV, risking failure as the afterload escalates.^210–214^ Acute pulmonary embolism (PE) abruptly obstructs pulmonary arteries, spiking RV pressure and impairing cardiac output, often culminating in acute RV failure if untreated.^215^ In COVID-19, SARS-CoV-2-driven pneumonia and ARDS disrupt alveolar integrity, elevating pulmonary arterial pressure and impairing oxygenation, straining the heart and inducing myocardial injury or arrhythmias, as evidenced by troponin elevation in severe cases.^216^ Immune activation compounds this; lung inflammation recruits macrophages and neutrophils, releasing ROS and proteases (e.g., elastase) that damage cardiac tissue, amplifying systemic inflammation and contributing to multiorgan dysfunction. Hypoxia resulting from lung injury triggers hypoxia-inducible factor-1α (HIF-1α), which upregulates endothelin-1, further constricting pulmonary vessels and stressing the RV.^217–219^

Heart–lung cross-talk drives diverse diseases. For example, intermittent hypoxia and ITP fluctuations in sleep apnea provoke LV dysfunction, systemic hypertension, and atrial fibrillation via SNS overdrive and RAAS activation.^144,145^ Sepsis, with its cytokine storm (e.g., IL-1β, IL-18) and hemodynamic instability, disrupts heart‒lung synchrony, often precipitating acute HF and ARDS, with gut-derived LPS amplifying the inflammatory cascade.^183,184^ Cystic fibrosis (CF) links chronic lung infection to RV strain, whereas the paraneoplastic effects of lung cancer—e.g., ectopic ACTH secretion—can induce cardiac stress via cortisol excess.^220,221^ Neurohumoral regulation, via SNS acceleration of heart rate and endothelial nitric oxide (NO) modulation of PVR, tightly couples these systems, with disruptions manifesting as exertional dyspnea or cor pulmonale.^222,223^

The heart‒lung axis is a therapeutic target for various systemic diseases. Beta-blockers (e.g., bisoprolol) reduce SNS overdrive, lowering heart rate and RV strain in HF patients and easing pulmonary workload in COPD patients, with clinical trials showing improved ejection fraction.^211,224^ Acetylcholinesterase inhibitors increase parasympathetic tone, counteracting SNS effects and stabilizing cardiopulmonary function, particularly in HFpEF.^225^ Anti-inflammatory therapies, such as IL-6 inhibitors (e.g., tocilizumab) or corticosteroids, mitigate cytokine-driven damage, effectively treating ARDS and severe COVID-19 by reducing lung edema and myocardial stress.^226,227^ Optimized mechanical ventilation—low tidal volumes and positive end–expiratory pressure (PEEP)—minimizes barotrauma and hemodynamic strain in ARDS, preserving cardiac output and oxygenation, as validated in intensive care unit (ICU) cohorts.^228,229^ Emerging strategies target endothelial function; NO-enhancing agents (e.g., inhaled NO) or prostacyclin analogs (e.g., epoprostenol) reduce PVR in PH patients and improve RV performance and V/Q matching, with preclinical data showing reduced RV fibrosis.^230^ Phosphodiesterase-5 inhibitors (e.g., sildenafil) increase NO signaling, alleviating PH and RV strains.^231,232^ Lifestyle interventions, such as aerobic exercise, increase cardiopulmonary reserve, increase VO₂ max, and mitigate early disease progression, whereas smoking cessation curbs COPD exacerbation and cardiac risk.^213,233,234^

In conclusion, in addition to its general role in sustaining oxygenation and circulation, the heart‒lung axis provides vital insight into hemodynamic and inflammatory interdependence. Its regulation involves intricate neurohumoural signals and immune cascades that shape cardiopulmonary resilience. Understanding these mechanisms paves the way for precision interventions to prevent and mitigate cardiopulmonary collapse.

#### Heart‒liver cross-talk

##### Mechanisms of heart‒liver cross-talk

The heart‒liver axis integrates cardiovascular and hepatic functions through circulatory, neural, and endocrine pathways, sustaining metabolic and hemodynamic homeostasis. The heart delivers oxygenated blood to the liver via the hepatic artery, complementing nutrient-rich portal vein inflow from the gastrointestinal tract, while the liver metabolizes substrates and returns blood to the heart through the hepatic veins. This bidirectional cross-talk, which is essential for lipid metabolism, detoxification, and systemic balance, is disrupted in cardiometabolic diseases, revealing their interdependence.^26,154,235–238^

##### From dysfunction to targeted interventions

Cardiac dysfunction has profound hepatic effects. In HF, reduced cardiac output causes hepatic congestion by impairing venous outflow, leading to centrilobular necrosis, fibrosis, and elevated transaminases, which are hallmarks of cardiohepatic syndrome.^239^ In HF with preserved ejection fraction (HFpEF), elevated right atrial pressure heightens portal vein pressure, mimicking cirrhosis and fostering portal hypertension.^240,241^ Acute cardiogenic shock triggers hypoxic hepatitis, where severe hypoperfusion starves hepatocytes of oxygen, rapidly spiking alanine aminotransferase (ALT) and aspartate aminotransferase (AST).^242–244^ Inflammation amplifies this interplay; MI or HF release proinflammatory cytokines (e.g., IL-6 and TNF-α) into the circulation, inducing hepatic stellate cell activation and lipid dysregulation and accelerating metabolism-associated fatty liver disease (MAFLD).^235,236^ The liver’s cholesterol synthesis, when unchecked, feeds back to the heart, depositing lipids in coronary arteries, hastening atherosclerosis, and increasing cardiovascular risk.^245,246^ Oxidative stress, driven by reactive oxygen species (ROS) from the ischemic myocardium, further compromises hepatic microcirculation.^247^

Liver pathology, in turn, imposes significant cardiac burdens. Cirrhosis elevates portal hypertension, increasing systemic vascular resistance and cardiac preloading, which fosters cirrhotic cardiomyopathy—marked by diastolic dysfunction, prolonged QT intervals, and blunted contractile responses to stress.^235^ In MAFLD, hepatic steatosis and insulin resistance disrupt lipid homeostasis, releasing triglycerides and low-density lipoproteins (LDLs) that promote coronary plaque formation and HF risk.^248,249^ Acute liver failure unleashes a torrent of inflammatory mediators (e.g., IL-1β and ammonia), triggering myocardial inflammation, oxidative damage, and arrhythmias.^250^ Impaired detoxification in liver disease allows uremic toxins and metabolic byproducts to accumulate, decreasing cardiac function and exacerbating systemic stress. Neuroendocrine pathways regulate this axis; mineralocorticoid receptor (MR) activation in hepatocytes modulates cardiac repair post-MI via fibroblast growth factor 21 (FGF21), whereas an upstream IL-6–STAT3 pathway suppresses MR expression, increasing cardioprotection—a dynamic liver–to-heart feedback loop.^251^

Many therapeutic strategies have been developed to target the heart‒liver axis. Beta-blockers counteract SNS-driven cardiac strain and hepatic glycogenolysis, improving HF outcomes and reducing portal pressure in cirrhosis.^252,253^ Anti-inflammatory agents, such as IL-6 inhibitors, dampen cytokine storms, halting MAFLD progression and myocardial damage, particularly in acute settings such as sepsis.^254^ Metabolic modulators offer dual benefits: glucagon-like peptide-1 (GLP-1) receptor agonists enhance insulin sensitivity, reducing hepatic lipid accumulation and HF risk, whereas SGLT2 inhibitors lower blood glucose, promote natriuresis, and decrease hepatic steatosis, protecting both organs in diabetes and HF.^255–257^ Spironolactone, an MR antagonist, upregulates hepatic FGF21 to increase post-MI repair, as validated in mouse models where hepatocyte STAT3 deficiency worsens outcomes.^258^ Nanotechnology-based liver-targeted drugs modulate metabolites such as LPC, fine-tuning lipid signaling to mitigate atherosclerosis. Lifestyle interventions, such as the Mediterranean diet—rich in omega-3 fatty acids and antioxidants—optimize lipid profiles, reduce inflammation, and bolster cardiovascular resilience.^259^ Exercise increases hepatic FGF21 production, supporting cardiac metabolism and repair.^260,261^

The heart‒liver axis integrates circulatory function with metabolic precision, serving as a cornerstone of systemic health. Its regulation spans endocrine signaling and inflammatory interactions, shaping cardiometabolic dynamics. Unraveling these connections offers a pathway for developing integrated therapies for cardiometabolic diseases.

### Gut

The gastrointestinal (GI) system, commonly referred to as the gut, is a multifaceted organ system that orchestrates the digestion of food, absorption of nutrients, and elimination of waste through a symphony of enzymatic secretions and coordinated muscular activity. In addition to its traditional role in nutrient processing, the gut harbors a thriving ecosystem of microbial communities—collectively known as the gut microbiota—that play a pivotal role in health by fermenting indigestible dietary fibers and endogenous mucus into bioactive metabolites, such as SCFAs, vitamins, and secondary bile acids. These microbial allies extend the gut’s influence far beyond digestion, contributing to metabolic regulation, immune system maturation, and neurological signaling.^262–268^ Advances in epidemiology, physiology, and omics-based research have redefined the gut as a central immunological, metabolic, and neurological hub, intricately linked to systemic homeostasis through its microbial inhabitants and their molecular products.^269^

From the organ cross-talk perspective, the gut has now been appreciated as a critical nexus in the interorgan network, dynamically interacting with distant systems such as the brain, liver, kidneys, lungs, and skin via circulatory, lymphatic, and neural pathways. The gut microbiota and its metabolites act as key mediators in these dialogs, modulating organ function and responding to physiological cues from across the body. For instance, microbial-derived SCFAs can bolster intestinal barrier integrity and influence hepatic lipid metabolism, whereas gut dysbiosis, marked by shifts in microbial composition, can trigger systemic inflammation that impacts renal filtration or pulmonary immunity.^270–275^ Conversely, organ dysfunction, such as liver cirrhosis or chronic kidney disease, can reshape the gut microbial landscape, perpetuating a feedback loop of pathology. This bidirectional interplay underscores the role of the gut as both a responder and a driver in multiorgan networks, with implications for a wide array of diseases.^276,277^

Given its central position and far-reaching effects, the gut has emerged as a research hotspot, illuminating how its dysfunction contributes to intra-abdominal and extra-abdominal disorders, from inflammatory bowel disease to neurodegenerative conditions and cardiometabolic syndromes.^278^ The following subsections delve into specific examples of gut-centered cross-talk, with the liver, kidneys, and lungs, detailing the molecular mechanisms, microbial contributions, and clinical relevance of these interactions. By exploring these axes, we aim to highlight the transformative potential of the gut as a therapeutic target and its pivotal role in bridging microbial activity with systemic health, offering new avenues for understanding and managing complex diseases.

#### Gut‒liver cross-talk

##### Mechanisms of the gut‒liver cross-talk

The gut‒liver axis orchestrates a vital interplay between intestinal and hepatic functions, mediated by the gut microbiota and its metabolites, which regulate metabolism, immunity, and inflammation. Anatomically linked via the portal vein, the gut delivers nutrients, microbial products, and toxins to the liver, whereas the liver secretes bile acids (BAs) into the intestine via the bile duct, sculpting microbial composition and intestinal homeostasis.^276^ This bidirectional cross-talk, which is fundamental to nutrient processing and immune surveillance, occurs in liver diseases, revealing their interdependence (Fig. 5).

##### From dysfunction to targeted interventions

Gut dysfunction exerts profound hepatic effects through microbial dysbiosis. SCFAs, derived from Bacteroidetes and Firmicutes fermentation of dietary fibers, reinforce intestinal barrier integrity by stimulating goblet cell mucin production and upregulating IL-18 and GLP-1 in epithelial cells. Transported via the portal vein, SCFAs increase hepatic β-oxidation and inhibit lipogenesis, mitigating MAFLD.^279–281^ Conversely, dysbiosis shifts this balance; alcohol-producing bacteria (e.g., *Klebsiella pneumoniae*) increase the level of endogenous ethanol, disrupting mitochondrial integrity and accelerating MAFLD.^282–284^ LPS, an outer membrane component of gram-negative bacteria, activates hepatic Toll-like receptor 4 (TLR4) on Kupffer cells, sparking a cascade of proinflammatory cytokines (e.g., TNF-α, IL-6, and IL-1β) that drive inflammation, stellate cell activation, and fibrosis in nonalcoholic steatohepatitis (NASH) and alcoholic liver disease (ALD).^285^ Tryptophan metabolites, such as indole and indole-3-acetic acid, activate aryl hydrocarbon receptor (AhR) to suppress lipogenic genes (e.g., SREBP1c), but microbial imbalance upregulates SREBP2 via tryptophan metabolism, fueling hepatocellular carcinoma (HCC).^286^ Branched-chain amino acids (BCAAs) from dysbiotic Bacteroides stercoris impair the hepatic tricarboxylic acid cycle, exacerbating mitochondrial dysfunction in MAFLD.^287^

Liver dysfunction reciprocally disrupts the gut. Cirrhosis impairs BA synthesis by downregulating CYP7A1, reducing bile flow, and altering microbial diversity, which hinders intestinal stem cell renewal and weakens barrier function, exacerbating IBD.^288,289^ In MAFLD and NASH, hepatic lipid overload and oxidative stress release proinflammatory mediators (e.g., IL-6 and ROS) into the portal circulation, increasing gut permeability and inflammation. This facilitates the translocation of microbial products such as LPS and trimethylamine N-oxide (TMAO), which return to the liver, perpetuating hepatic injury and fibrosis.^290,291^ Bile acid receptors, farnesoid X receptor (FXR) and TGR5, mediate this feedback loop; FXR activation bolsters barrier integrity and GLP-1 secretion, whereas TGR5 curbs inflammation via cAMP signaling—both of which are compromised in liver disease states such as primary sclerosing cholangitis (PSC).^292^

This axis underpins a spectrum of pathologies. In ALD, alcohol-induced dysbiosis increases LPS and ethanol translocation, accelerating hepatic fibrosis and steatosis via TLR4‒NF-κB signaling. Primary sclerosing cholangitis (PSC) and primary biliary cholangitis (PBC) reflect gut-derived immune insults, with microbial metabolites (e.g., TMAO and indoxyl sulfate) exacerbating cholestasis, bile duct injury, and systemic inflammation.^293–295^ HCC links to microbial tryptophan metabolism, upregulating AhR–SREBP2 and promoting oncogenic lipid synthesis and tumor growth.^296,297^ Systemic inflammation, fueled by cytokines (e.g., IL-6 and IL-17) and immune cells (e.g., dendritic cells (Tregs)), bridges the gut and liver, amplifying disease progression in sepsis, where gut barrier failure drives liver injury and multiorgan dysfunction.^298,299^

Therapeutic strategies target this cross-talk to restore equilibrium. Probiotics (e.g., Bifidobacterium and Lactobacillus) and prebiotics recalibrate microbiota, increasing SCFA production to reinforce the gut barrier and reduce hepatic inflammation, with clinical trials showing reduced AST–ALT levels in ALD and NASH.^300^ FXR agonists (e.g., obeticholic acid) increase BA signaling, improving lipid metabolism and barrier function in PBC and NASH patients, whereas TLR4 inhibitors block LPS-driven Kupffer cell activation, attenuating fibrosis in ALD patients and NASH patients.^301^ Novel microbial therapies include pentadecanoic acid from Parabacteroides distasonis, which restores gut barrier integrity and suppresses hepatic triglyceride synthesis in NASH, and sphingolipids from Bacteroides spp., which increase lipid oxidation via serine palmitoyltransferase.^302–304^ Fecal microbiota transplantation (FMT) recalibrates microbial ecosystems, showing promise in IBD, early HCC, and *Clostridioides difficile* infections by reducing pathogenic translocation.^305,306^ Dietary interventions, such as high-fiber or Mediterranean diets, promote beneficial metabolites (e.g., SCFAs and carotenoids), offering a practical adjunct to dampen inflammation and support liver health.^307,308^

The gut‒liver axis affects microbial ingenuity with hepatic resilience, channeling metabolic and immune dialogs via the portal vein. Probing its microbial‒metabolic nexus unveils a therapeutic frontier to disarm liver disease at its intestinal roots, harnessing microbial allies to recalibrate this vital axis.

#### Gut–kidney cross-talk

##### Mechanisms of the gut‒kidney cross-talk

The gut–kidney axis governs the dynamic interplay between intestinal and renal functions, which is mediated by blood circulation, lymphatic pathways, and microbial metabolites, orchestrating systemic homeostasis. The gut modulates kidney function by delivering microbial products via the bloodstream, whereas the kidneys influence gut physiology through hormonal cascades and metabolic waste clearance.^309^ This bidirectional cross-talk, pivotal for fluid balance, immune regulation, and metabolic harmony, falters in renal diseases, highlighting the role of the gut as a sentinel of kidney health and systemic inflammation (Fig. 6).

##### From dysfunction to targeted interventions

Gut dysfunction profoundly impacts the kidney through microbial dysbiosis. Beneficial metabolites such as SCFAs and vitamin K, produced by commensals (e.g., *Faecalibacterium prausnitzii*), confer nephroprotection; SCFAs activate renal GPR43 to suppress fibrosis, whereas vitamin K quells oxidative stress by increasing antioxidant defenses.^310,311^ Conversely, dysbiosis generates nephrotoxic metabolites—p-cresyl sulfate (pCS), indoxyl sulfate (IS), indole acetic acid (IAA), and TMAO—which impair the glomerular filtration rate (GFR) and tubular integrity.^312,313^ TMAO, for example, activates the NLRP3 inflammasome and skews macrophages toward M1 polarization, accelerating interstitial fibrosis and tubular atrophy in chronic kidney disease (CKD).^314^ LPS from gram-negative bacteria amplifies this process via TLR4 signaling, recruiting neutrophils and releasing IL-1β, exacerbating renal inflammation.^315^ In hyperuricemia, purine-rich diets shift the microbiota toward uric acid producers (e.g., Clostridium spp.), overwhelming renal excretion and triggering crystal formation and fibrosis—effects countered by probiotics such as Lactobacillus rhamnosus, which restore SCFA levels and barrier function.^316^ Dietary protein overload further tips this balance, with gut-derived uremic toxins such as pCS accumulating in renal tubules, hastening CKD progression.^317,318^

Kidney dysfunction reciprocally reshapes the gut. In CKD, uremic toxins (e.g., urea, creatinine, and uric acid) accumulate, altering the gut’s biochemical milieu and fostering dysbiosis—evidenced by reduced Bifidobacterium and Lactobacillus and increased Prevotella and Clostridium IV.^319^ This disrupts tight junctions, heightening bacterial translocation and systemic inflammation, as observed in end-stage renal disease (ESRD), where circulating LPS drives cytokine storms (e.g., TNF-α and IL-6). Renal hormones modulate this axis; 1,25-dihydroxyvitamin D3 (1,25(OH)₂D₃) deficiency weakens mucosal immunity, whereas erythropoietin (EPO) decline in CKD impairs gut hypoxia responses, exacerbating dysbiosis. RAAS overactivation, via angiotensin II, promotes gut inflammation and permeability, compounding barrier breaches.^320,321^ Lymphatic dysfunction in kidney injury—marked by increased lymph flow and vessel dilation—enhances the transport of lipids, lipoproteins (e.g., isolevuglandin-modified apoAI [IsoLG-apoAI]), cytokines (e.g., IL-17), and secretory IgA (SIgA), amplifying systemic immune activation and gut-derived inflammation and further taxing renal function.

This axis fuels a spectrum of renal pathologies. In CKD, gut-derived TMAO and IS correlate with fibrosis progression and mortality risk, with Prevotella and Parabacteroides serving as early diagnostic biomarkers detectable before proteinuria.^322,323^ IgA nephropathy stems from gut dysbiosis, where aberrant IgA1 glycosylation—driven by microbial enzymes—forms immune complexes that deposit in glomeruli, igniting inflammation, mesangial proliferation, and fibrosis.^324^ Kidney stone disease is linked to oxalate-producing microbiota depletion (e.g., Oxalobacter formigenes), increased urinary oxalate, and stone formation, compounded by dysbiotic shifts in Bacteroides.^325–327^ Acute kidney injury (AKI) breaks gut barriers via systemic inflammation, with LPS and cytokine surges (e.g., IL-1β) worsening renal tubular damage and delaying recovery. Sepsis intensifies this vicious cycle, with gut-derived toxins and cytokines (e.g., IL-6 and TNF-α) synergizing to precipitate acute renal failure and multiorgan collapse, which is often resistant to conventional dialysis.^328^

Therapeutic strategies target this axis to restore harmony. Probiotics (e.g., Lactobacillus casei and *Bacteroides fragilis*) increase SCFA production, reduce LPS translocation and inflammation, and slow CKD and AKI progression in preclinical models—L. casei boosts nicotinamide levels, tempering tubular injury.^329^ Prebiotics, such as inulin, shift the microbiota toward SCFA producers, lowering oxalate and uremic toxins, as validated in kidney stone prevention trials.^330,331^ TMAO inhibitors (e.g., ALG-05, which targets tryptophan indole lyase) curb IS production, easing fibrosis, whereas tight junction enhancers (e.g., chitosan oligosaccharide [COS]) activate intestinal CaSR/AMPK pathways, reducing systemic inflammation and oxidative stress in prediabetes, with COS also balancing mTOR-autophagy dynamics in renal cells.^332^ Fecal microbiota transplantation (FMT), including washed microbiota transplantation (WMT), restores microbial equilibrium, expels toxins, and improves the glomerular filtration rate (GFR) in CKD patients. Dietary interventions—high-fiber or Mediterranean diets—foster SCFA-producing genera (e.g., Akkermansia), dampening inflammation and supporting renal repair. Berberine inhibits tyrosine–p-cresyl pathways, reducing pCS and increasing butyrate, offering dual gut–renal protection, with preclinical data showing alleviated fibrosis and increased microbiota diversity.^333,334^

The gut–kidney axis involves microbial orchestration with renal filtration, which threatens systemic health through circulatory and lymphatic conduits. Decoding its microbial–metabolic tapestry unveils a restorative arsenal to shield kidneys from gut-driven assaults, leveraging microbial symbiosis to fortify renal resilience.

#### Gut‒lung cross-talk

##### Mechanisms of the gut–lung cross-talk

The gut‒lung axis orchestrates a bidirectional dialog between the gastrointestinal tract and respiratory system, integrating microbial metabolites, immune responses, and neural signals to regulate pulmonary and intestinal homeostasis. Although anatomically distant, the gut shapes lung immunity through circulating microbial products delivered via the bloodstream and lymphatic routes, whereas lung pathology reciprocally alters the gut microbiota composition and barrier integrity via systemic inflammation and oxidative stress.^335–337^ This intricate cross-talk, which is pivotal for respiratory and immune balance, has emerged as a key player in health and disease, as revealed by multiomics profiling, microbiome sequencing, and preclinical models dissecting microbial‒immune interactions.

##### From dysfunction to targeted interventions

Gut dysfunction profoundly influences lung health through microbial metabolites and immune modulation. SCFAs, such as butyrate and propionate—produced by Prevotella, Bifidobacterium, and other fiber-fermenting bacteria—exert potent anti-inflammatory effects; butyrate inhibits type 2 innate lymphoid cells (ILC2s) in the lungs, reducing airway inflammation in allergic models, whereas propionate attenuates dendritic cell (DC) activation, mitigating Th2-driven allergic airway disease.^338–340^ Tryptophan derivatives, such as deaminated tryptophan (DAT), increase type I interferon responses via gut–lung signaling, bolstering resistance to influenza viruses, as seen in mouse models.^341,342^ Phenol sulfate (PCS), derived from microbial L-tyrosine metabolism, suppresses EGFR-enhanced TLR4/NF-κB signaling, curbing allergic inflammation. Conversely, dysbiosis elevates the levels of proinflammatory metabolites; TMAO correlates with pulmonary arterial hypertension (PAH) severity, impairing endothelial relaxation and increasing pulmonary vascular resistance, whereas succinate—which accumulates after intestinal ischemia/reperfusion—activates alveolar macrophages SUCNR1 and the PI3K/AKT/HIF-1α pathway, triggering acute lung injury (ALI).^343,344^ Gut-derived immune cells, such as ILC2s, migrate to the lungs via circulation, amplifying pulmonary inflammation in asthma and COPD, with their activity modulated by microbial signals such as SCFAs and bile acid derivatives.^345^

Lung pathology, in turn, reshapes the gut ecosystem. COPD and idiopathic pulmonary fibrosis (IPF) induce hypoxia and systemic inflammation, reducing the abundance of Lactobacillus and Bifidobacterium in the gut while increasing the abundance of Streptococcus and Actinobacteria, reflecting disease progression and correlating with fecal metabolite shifts (e.g., reduced SCFA levels). In COVID-19, SARS-CoV-2 infection depletes beneficial bacteria (e.g., Lactobacillus), elevating Clostridium and Haemophilus, which are associated with severe pneumonia, cytokine storms (e.g., IL-6 and IL-1β), and increased mortality risk.^346,347^ Asthma’s chronic inflammation fosters early-life microbial imbalances, with Streptococcus, Bacteroides, and Clostridium enrichment linked to increased airway hyperresponsiveness, whereas reduced Faecalibacterium predicts childhood asthma risk.^348,349^ Lung-derived inflammation—by circulating cytokines (e.g., TNF-α and IL-17) and oxidative stress markers—compromises gut barrier integrity by disrupting tight junctions and increasing permeability and microbial translocation, which feeds back to exacerbate pulmonary inflammation and injury. Neurohumoral pathways amplify this loop; stress-induced SNS activation alters the gut microbial composition via vagal signaling, whereas pulmonary hypoxia triggers gut hypoxia responses, further skewing the microbial balance and systemic immunity.

This axis underpins a broad spectrum of respiratory diseases. In asthma, microbial dysbiosis (e.g., reduced Veillonella) is correlated with airway remodeling, whereas IPF gut dysbiosis (e.g., elevated Staphylococcus) parallels lung Bacteroides overgrowth, activating TLR2/TLR4 via outer membrane vesicles and driving IL-17B-mediated fibrosis.^350,351^ The fecal Streptococcus dominance of COPD patients has emerged as a diagnostic marker reflecting chronic inflammation and oxidative stress. Nontuberculous mycobacterial (NTM) infections worsen Enterobacteriaceae colonization, reducing the number of alveolar macrophages and dendritic cells, whereas influenza susceptibility spikes with antibiotic-induced microbiota depletion, impairing TLR7 signaling and antiviral defenses.^352^ Sepsis couples gut dysbiosis with ALI, amplifying lung injury via IL-18 precursor release and microbial translocation, which are often resistant to standard interventions.

Therapeutic strategies involve recalibrating this cross-talk to restore balance. Probiotics (e.g., Butyricicoccus pullicaecorum and Lactobacillus casei) increase ω-3 fatty acid (e.g., 18-hydroxyeicosapentaenoic acid [18-HEPE]) and IFN-λ expression in lung epithelial cells, enhancing antiviral immunity against influenza and SARS-CoV-2, with L. casei reducing airway inflammation in asthma models via SCFA-mediated Treg induction.^353,354^ Prebiotics such as dietary fiber increase SCFA producers (e.g., Bifidobacterium and Faecalibacterium), alleviating chronic obstructive pulmonary disease (COPD), cystic fibrosis, and asthma symptoms by modulating systemic immunity.^354^ TMAO inhibitors reverse PAH progression, restoring vascular tone and reducing right ventricular strain, whereas exogenous SCFA supplementation (e.g., propionate) curbs allergic inflammation by enhancing Treg differentiation and IL-10 production. Fecal microbiota transplantation (FMT) restores gut microbial diversity, improving COPD and asthma outcomes in preclinical trials and showing potential in NTM infections by increasing alveolar defenses via L-arginine enrichment and macrophage activation.^355^ Anti-inflammatory agents (e.g., IL-6 inhibitors) mitigate sepsis-induced lung–gut damage by suppressing cytokine storms, whereas corticosteroids temper ARDS inflammation, preserving lung function.^254^ Dietary interventions, such as high-fiber or Mediterranean diets, increase microbial production of SCFAs and polyphenols (e.g., quercetin), supporting lung repair, reducing asthma severity, and bolstering epithelial integrity.^356,357^ Precision approaches, such as microbial enzyme inhibitors targeting succinate production, aim to disrupt proinflammatory cascades, offering future therapeutic promise validated in ischemia–reperfusion models.^358^

The gut–lung axis threads microbes into pulmonary resilience, weaving a distant yet profound symbiosis. Decoding its metabolic-immune choreography unveils a therapeutic symphony to mute respiratory discord through intestinal harmony.

## Methods and technologies

The human body is a complex network of interconnected organs that communicate through intricate physiological, metabolic, and molecular pathways. Understanding these interorgan interactions is crucial for unraveling the mechanisms underlying health and disease. In recent years, advancements in technology and methodology have revolutionized our ability to study these connections across multiple scales—from macroscopic anatomical structures to microscopic molecular activities. This chapter provides a comprehensive overview of the key approaches and technologies used to investigate interorgan interactions, including traditional anatomical studies, cutting-edge molecular imaging techniques, disease-specific models, animal experiments, organoid systems, molecular-level analyses, and artificial intelligence-driven methodologies. By integrating these diverse tools, researchers are gaining unprecedented insights into the dynamic relationships between organs, paving the way for more precise diagnostics and targeted therapies.

### Traditional and imaging-based approaches

Traditional physiology and pathology methods are based on anatomical studies of the human body at the macro level. However, with advancements in molecular biology and biotechnology, researchers can now explore the structure and function of the body at the micro level via molecular imaging techniques. Among these techniques, magnetic resonance imaging (MRI) and computed tomography (CT) are primarily used to visualize the anatomical structures of organs and their spatial relationships.^359,360^ In contrast, positron emission tomography (PET) and single-photon emission computed tomography (SPECT) provide insights into molecular-level interactions and metabolic connectivity between organs.^361–363^

These imaging technologies have been instrumental in revealing both the physiological status of individual organs and the complex interactions among multiple organ systems. For example, MRI has been widely used in large-scale studies to uncover interorgan relationships. In a study using UKB cohort data, researchers analyzed MRI data from ~30,000 individuals and derived imaging indices for the heart, brain, and liver via standardized protocols. Through multivariable linear regression and three-organ modeling analysis, they identified associations among cardiac function, brain structure, and liver metabolism, highlighting physiological-level interconnections among these organs.^16^

Functional magnetic resonance imaging (fMRI), a specialized application of magnetic resonance imaging (MRI), has been particularly valuable in studying functional connections between the brain and other organs, such as within the brain‒gut axis. In studies of patients with IBS, fMRI has been used to monitor neural activity during rectal balloon distension and evaluate the activation of diffuse noxious inhibitory controls (DNICs). In healthy controls, DNIC activation reduced pain scores, whereas this effect was absent in IBS patients, with distinct patterns of brain region activation observed between the two groups. These findings suggest that the functional connections of the brain–gut axis differ significantly between healthy individuals and IBS patients.^71^ Furthermore, another fMRI study revealed that IBS patients exhibited stronger activation of the anterior cingulate cortex under painful stimuli, along with higher reported pain intensity, indicating abnormal pain sensitivity within the brain–gut axis.^364^

PET imaging has emerged as a powerful tool for exploring metabolic connectivity between organs. In a study of healthy individuals, researchers employed PET technology with ^18^F-FDG as a tracer to comprehensively capture metabolic data from 16 subjects. The PET/CT scanning device accurately recorded functional imaging information during metabolic activities, revealing not only the morphological and structural details of organs but also their metabolic activity and interorgan correlations. Through in-depth analysis, the study demonstrated that the liver, kidneys, and brain presented the strongest metabolic connectivity.^365^

Overall, these molecular imaging techniques—ranging from structural MRI and CT to functional fMRI and metabolic PET—have significantly expanded our understanding of the body’s complex interorgan relationships. By providing detailed insights into anatomical, functional, and metabolic interactions, they hold immense potential for advancing disease diagnosis and therapeutic strategies.

### Disease models and animal studies

Understanding the connections between organs can be significantly advanced through the study of specific diseases, which often serve as natural models of organ cross-talk. By examining how diseases disrupt normal physiological interactions, researchers can uncover the underlying mechanisms of organ communication and identify potential therapeutic targets. For example, acute kidney injury (AKI) has been extensively used to study the relationship between the kidneys and other organs, such as the heart and lungs. AKI-induced systemic inflammation and metabolic dysregulation can lead to distant organ damage, highlighting the central role of the kidneys in maintaining homeostasis.^366–368^ Similarly, liver-related encephalopathy provides a valuable model for investigating liver‒brain cross-talk. Under these conditions, the accumulation of neurotoxins due to impaired liver function disrupts brain activity, offering insights into the metabolic and signaling pathways linking these two organs.^78^

A compelling example bridging developmental and disease perspectives is the patent ductus arteriosus (PDA), which illustrates heart–lung cross-talk across life stages. During fetal development, the ductus arteriosus facilitates blood shunting from the pulmonary artery to the aorta, bypassing nonfunctional lungs. However, persistent patency after birth can lead to excessive pulmonary blood flow, pulmonary hypertension, or heart failure, demonstrating how a developmentally adaptive structure can precipitate pathological organ interactions if unresolved. Stroke, a leading cause of disability worldwide, has been instrumental in studying heart–brain interactions. Research has shown that stroke can trigger cardiac dysfunction through mechanisms such as autonomic nervous system dysregulation and systemic inflammation, underscoring the bidirectional nature of heart–brain cross-talk.^119^ More recently, COVID-19 has emerged as a unique model for exploring lung–gut interactions. The virus not only causes respiratory distress but also disrupts the gut microbiota and intestinal barrier function, leading to systemic inflammation and multiorgan complications.^369^ Additionally, the immune system plays a critical role in mediating organ cross-talk, particularly in autoimmune diseases. Conditions such as rheumatoid arthritis and systemic lupus erythematosus are associated with cardiovascular complications, including atherosclerosis and myocardial infarction.^370,371^ These diseases illustrate how chronic inflammation and immune dysregulation can disrupt the normal function of distant organs, further emphasizing the interconnectedness of the body’s systems.

Studying specific diseases provides a powerful lens through which to examine the intricate interactions and connections between organs. These disease models not only reveal the physiological and pathological mechanisms underlying organ cross-talk but also offer opportunities for developing targeted interventions to restore normal function.

Animal experiments constitute another cornerstone of research into organ axes and interorgan interactions. By leveraging the genetic and physiological similarities between animals and humans, researchers can manipulate specific variables and observe their effects on organ systems in a controlled environment. For example, mouse studies on the gut–brain axis have been pivotal in uncovering the role of SCFAs in microbiota–gut–brain signaling.^372^ Similarly, mouse models of AKI have revealed critical insights into cardiorenal syndrome, demonstrating that kidney-derived IL-33 signals directly to cardiomyocytes via its receptor ST2L, driving cardiac hypertrophy and cardiomyopathy. These findings underscore the utility of animal models in elucidating molecular pathways of organ cross-talk and identifying potential therapeutic targets for multiorgan diseases.^373^

Overall, animal experiments provide a versatile and powerful platform for studying organ axes and uncovering the physiological mechanisms of interorgan interactions. By combining disease models with animal studies, researchers can bridge the gap between basic science and clinical applications, ultimately advancing our understanding of human health and disease.

### Organoids and multiorgan systems

Organoid research has emerged as a cutting-edge field driven by technological advancements that provide a highly controllable platform for studying organ-level interactions. By cultivating organoids resembling various tissues, researchers can simulate realistic physiological and biochemical processes, enabling in-depth exploration of interorgan communication and disease pathology. For example, a microfluidic multiorganoid system constructed from human induced pluripotent stem cells (hiPSCs) has successfully modeled the liver–islet axis, replicating glucose regulation processes and offering a novel platform for studying the pathology of type 2 diabetes.^374^ In addition, a group from Columbia University developed a multiorgan chip with four chambers containing hiPSC-derived heart, liver, bone, and skin, connected by an endothelial barrier and vascular flow. This device was successful not only in recapitulating pharmacokinetic and pharmacodynamic profiles but also in modulating prolonged organ damage induced by space radiation.^375^

Similarly, the development of heart–kidney connected organoids has opened new avenues for investigating cardiorenal syndrome. By integrating coculture techniques, microfluidic platforms, and 3D printing technology, researchers can precisely observe the mutual effects of cardiac and renal pathologies in a controlled environment. These models have revealed critical insights into the bidirectional cross-talk between the heart and kidneys, such as the role of inflammatory cytokines and metabolic dysregulation in disease progression.^165^

Organoid models have broad application prospects in multiorgan disease research, drug development, and personalized medicine. They not only provide a more ethical alternative to animal experimentation but also enable researchers to study human-specific physiological and pathological processes with greater precision and efficiency. As organoid technology continues to advance, it has revolutionized our understanding of interorgan interactions and accelerated the development of targeted therapies for complex diseases.

### Molecular and omics-level insights

Molecular and omics-level research provides a powerful lens for uncovering the intricate connections between different organs, offering insights into the dynamic interactions that underlie physiological and pathological processes. By analyzing metabolites, immune molecules, and hormones, researchers can elucidate the molecular mechanisms of organ cross-talk and identify potential therapeutic targets. Metabolomics, for example, has revealed how changes in the metabolic profiles of one organ can influence the functions of other organs. For example, bilirubin, a liver metabolite, regulates the liver–gut axis by interacting with the intestinal microbiota, demonstrating how liver metabolism influences gut function.^376^ Similarly, neuroactive metabolites such as gamma-aminobutyric acid and short-chain fatty acids, produced by the gut microbiota, modulate brain function and emotional regulation, illustrating the metabolic cross-talk within the gut–brain axis.^377^

Epigenetic mechanisms further enrich this molecular perspective by revealing how environmental and physiological signals dynamically regulate organ interactions. Epigenome-wide association studies (EWASs) have revealed stress-induced DNA methylation changes, such as hypermethylation of the NR3C1 (glucocorticoid receptor) gene in the central amygdala, which modulates visceral pain sensitivity in the gut‒brain axis. These epigenetic modifications, alongside histone acetylation at stress-responsive gene promoters such as corticotropin-releasing hormone (CRH), illustrate how the epigenome integrates metabolic and immune signals to influence organ cross-talk, particularly under chronic stress conditions.^378^ Immune molecules such as cytokines and chemokines can also provide information about the cross-talk between organs, particularly in the context of inflammation and the immune response.^379–381^ Furthermore, hormones such as insulin, cortisol, and thyroid hormones can indicate the interdependence between different organs, such as the pancreas, adrenal glands, and thyroid gland.^382,383^ By integrating molecular and omics-level data, researchers can map complex networks of organ cross-talk and uncover novel biomarkers for disease diagnosis and treatment. This approach not only deepens our understanding of human physiology but also paves the way for the development of targeted therapies that address the root causes of multiorgan diseases.

### Artificial intelligence and integrative approaches

Artificial intelligence (AI) has revolutionized the study of organ interactions by enabling the analysis of complex, high-dimensional datasets with unprecedented precision and efficiency. AI-powered algorithms, particularly in machine learning and deep learning, are now widely applied to medical imaging data, such as MRI, CT, and PET scans, to automatically detect patterns, segment organ boundaries, and predict interorgan dysfunctions on the basis of subtle changes in imaging data.^384–388^ These techniques not only provide faster and more accurate analyses than traditional methods do but also enhance our understanding of organ interactions and facilitate early disease detection.

In addition to imaging, AI-driven methodologies are transforming the analysis of big data to uncover complex organ connections. AI models excel at processing large-scale, heterogeneous datasets, such as electronic health records (EHRs) and multiomics data, revealing previously undetectable patterns of interorgan cross-talk.^389^ For example, the open-source framework *ehrapy* has been developed to standardize the analysis of EHR data, offering modular pipelines for data extraction, quality control, patient clustering, survival analysis, and trajectory inference.^390^ This framework has been successfully applied to stratify patients with unspecified pneumonia into finer-grained phenotypes, identify biomarkers linked to survival differences, and quantify the effects of medication classes on clinical outcomes, demonstrating the potential of AI to uncover complex interorgan relationships.

AI also plays a critical role in predictive modeling, where advanced algorithms analyze clinical, imaging, and multiomics data to forecast disease trajectories or predict the response of multiorgan systems to specific treatments.^391–394^ By integrating data from diverse sources, AI-driven approaches have identified novel biomarkers and therapeutic targets for conditions such as cancers, heart failure, hypertension, and chronic kidney disease.^395–399^

Furthermore, AI-enhanced organoid models and organ-on-a-chip systems are providing deeper insights into interorgan interactions.^400,401^ These systems enable real-time monitoring and analysis of organ functions, accelerating disease modeling and drug screening. For example, AI algorithms can analyze dynamic interactions within multiorgan systems, such as the liver‒heart axis, to identify key regulatory mechanisms and potential therapeutic interventions.

Overall, the integration of AI technologies is transforming our understanding of organ connections, offering new tools for personalized medicine and targeted therapies. By combining imaging, omics, and clinical data with advanced computational methods, AI is paving the way for more precise and effective approaches for disease diagnosis and treatment.

## Human multiorgan network and future perspectives

As research on the relationships between different organs and systems in the body continues to deepen, the construction and application of the whole-organ connection network holds vast promise, with signal transduction mechanisms serving as its central hub. The whole-organ connection network encompasses interactions and influences among major systems, such as the brain, heart, lungs, liver, kidneys, and gastrointestinal tract, all of which are dynamically regulated by internal physiological states and external environmental factors. Large-scale research projects, such as the UK Biobank (UKB) and Million Veteran Program, provide vast phenotypic datasets, laying a solid foundation for exploring deeper interorgan relationships.^402–405^

With the rapid development of technologies such as big data analysis, computer science, and artificial intelligence (AI), researchers can now systematically reveal the structure and function of the human organ network.^406–409^ In particular, AI has become a pivotal tool for analyzing the massive and complex datasets generated by these large-scale studies. Its computational power enables the integration and analysis of multimodal data, such as imaging, genomics, and clinical phenotypes, revealing complex patterns and relationships that traditional methods often miss.^410–412^ These advancements will drive the development of more accurate and personalized diagnostic, predictive, and therapeutic strategies for multiorgan diseases.

Future research must prioritize dynamic regulation within this network—how feedback loops adapt to stressors such as inflammation or metabolic imbalance, and how disruptions cascade across organs. Integrating these insights into predictive models will enhance early detection and prognosis, as seen in AI-driven stratification of heart failure or liver disease trajectories. Personalized interventions, leveraging organ network data, promise tailored therapies—e.g., modulating the gut microbiota to mitigate lung inflammation or targeting the RAAS to protect heart–kidney function—to shift healthcare toward systemic precision. To accelerate these efforts, we advocate for participation in collaborative initiatives such as the Human Phenome Project, a multicenter, cross-disciplinary program that integrates phenotypic data from diverse populations, fostering collaboration among clinicians, geneticists, and data scientists to deepen our understanding of organ cross-talk. Emerging technologies such as organoids offer a powerful platform for simulating multiorgan interactions in vitro, enabling researchers to study dynamic cross-talk under controlled conditions, such as liver–islet axis responses to metabolic stress, although challenges in replicating whole-body complexity remain. A significant unaddressed challenge is the construction of a comprehensive whole-organ network; however, progress is being made through initiatives such as the “Digital Human” project, which aims to digitally model systemic interactions, and achieving a fully integrated network with real-time physiological accuracy is still a frontier to conquer.

This multiorgan network, with signal transduction as its nexus, heralds a transformative frontier. By unraveling its complexities, we unlock novel diagnostics and therapies, offering hope for managing intricate diseases and advancing human health through a unified, network-driven lens.

Nevertheless, this review has certain limitations that merit attention. The discussion relies primarily on traditional organ-based data, with limited integration of network-driven analyses enabled by cutting-edge molecular, digital, and AI tools. Additionally, owing to length constraints, this review inevitably omits detailed discussions of many other major organs or systems (such as muscles and skeletal systems, despite their inclusion in tables). Similarly, a deeper understanding of dynamic, real-time interactions within the organ network remains a critical gap. These limitations elucidate directions for future research, particularly in the exploration of network-driven analyses and comprehensive interaction models.
